# Distributed Event-Driven Bayesian Search for Multi-UAV Systems with Spatially Correlated Targets

**DOI:** 10.3390/s26165189

**Published:** 2026-08-16

**Authors:** Dunbiao Niu, Peng Yi, Yiguang Hong

**Affiliations:** 1 Department of Control Science and Engineering, College of Electronics and Information Engineering, Tongji University, Shanghai 201804, China; dunbiaoniu@tongji.edu.cn (D.N.); yipeng@tongji.edu.cn (P.Y.); 2 Shanghai Research Institute for Intelligent Autonomous Systems, Tongji University, Shanghai 200092, China

**Keywords:** multi-UAV cooperative search, Bayesian inference, spatial correlation, event-triggered communication, distributed coordination

## Abstract

Rapid cooperative detection of stationary targets by multiple unmanned aerial vehicles (UAVs) is important in time-critical missions such as search and rescue. However, the online coordination of probabilistic inference, distributed communication, and detection–motion decisions under local information remain challenging when targets exhibit spatial correlations that existing methods typically neglect. To address this challenge, we develop a distributed event-driven Bayesian search framework for stationary, spatially correlated targets at unknown locations. The framework couples three components. A pairwise spatial model and a distance-dependent Neyman–Pearson detector yield a Bayesian belief update whose unclipped product form is order-invariant to event-processing sequence. A distributed selective flooding algorithm propagates only positive detection events, achieving finite-time event-set consensus over connected graphs while avoiding full-map exchange. A decoupled detection–motion planner exhausts high-belief cells within each UAV’s field of view before selecting a waypoint that balances surrogate detection probability against travel cost, with responsibility regions dynamically renegotiated among neighbors when local high-value cells are depleted. In numerical experiments, the proposed method achieved zero uncoordinated repeat detection in all simulations and significantly reduced first-discovery coverage relative to static-partition and no-communication baselines, while adapted external baselines required 90-fold and 6-fold larger communication payloads and had nonzero repeat-detection rates. The framework thus occupies a specific tradeoff point of zero revisit, sparse communication, and early discovery gain in scenes where targets span multiple UAV search regions.

## 1. Introduction

Unmanned aerial vehicles (UAVs) have been widely deployed in many missions, including target tracking and search [1,2], traffic surveillance [3], disaster management [4,5], and search-and-rescue operations [2,6]. In time-critical search missions, cooperation among multiple UAVs provides a scalable way to detect targets over uncertain regions. Each UAV observes a limited field of view (FoV), interrogates grid cells using an onboard detector, and exchanges detection evidence through wireless links. The resulting multi-UAV cooperative target search (MCTS) problem is challenging because the underlying path-optimization problem is NP-hard [7], the coordination architecture must remain distributed under dynamically evolving information, and onboard sensing, computation, and communication resources are limited. These challenges become more severe when targets exhibit spatial clustering, detection reliability depends on sensing geometry, and continuous exchange of high-dimensional belief maps becomes bandwidth-prohibitive. Moreover, neglecting spatial correlation has two concrete consequences. A positive detection at one cell provides no incentive to intensify the search in adjacent cells where additional targets are most probable. A negative observation cannot suppress belief in spatially coupled cells whose state is already predictable from earlier evidence. In both cases, the team wastes detection effort on areas whose status could have been inferred. Such clustering is common in practice. Vehicles in convoy, survivors near disaster shelters, and sensor nodes at regular spacing all exhibit spatial structures that a search policy should exploit [1,2].

Early studies often reduced cooperative search to an area-coverage problem and adopted preplanned deterministic patterns such as Zamboni or spiral search [8]. Geometry-based methods require the UAV team to maintain rigid formations, including parallel, spiral, or regular-polygon patterns, while sweeping the search area [9,10,11]. Although these methods are simple to implement, their fixed search patterns provide limited adaptability to evolving environments and nonuniform target distributions.

To improve adaptability, subsequent studies have developed real-time search frameworks in which UAVs repeatedly sense the environment, update local information, and choose motion-planning actions that optimize a search reward [12,13]. Model predictive control (MPC) is a widely used method for MCTS because it discretizes the mission region into a grid and solves a receding-horizon optimization problem involving target-existence probabilities and environmental uncertainty. However, centralized MPC incurs rapidly increasing communication and computation burdens as the team size grows [14,15]. Distributed MPC (DMPC) alleviates this difficulty by allowing each UAV to solve a local optimization problem using neighbor-exchanged information [16,17,18,19]. Heuristic solvers such as ant colony optimization [20], genetic algorithms [21], and differential evolution [22] are often embedded in such local decision problems. Voronoi partitions have also been combined with DMPC to preassign search regions and reduce inter-UAV conflicts [23]. Hollinger et al. proposed a distributed multirobot search framework that exchanges target-belief maps and planned paths, applies pointwise-minimum fusion upon reconnection, and coordinates actions through receding-horizon implicit coordination [24]. Hou et al. combined an importance map, centroidal Voronoi tessellation (CVT), auction-based task assignment, and receding-horizon predictive control (RHPC) for multi-UAV search [25]. Nevertheless, most DMPC-based methods rely on static prior information, such as initial target probability maps, and provide limited mechanisms for detection-driven redistribution of search responsibility. More broadly, distributed optimization [26,27] and resource-allocation methods provide powerful tools for networked agents with local objectives, local constraints, and limited communication [28]. However, their standard formulations are not tailored to event-driven Bayesian target search with spatially correlated beliefs and distance-dependent sensing. Secure and efficient UAV communication is an active complementary research area, with recent studies on physical-layer security exploiting reconfigurable-intelligent-surface control, collaborative beamforming, and reinforcement learning [29,30]. While these works strengthen the communication substrate, our focus is to reduce the search information that must traverse the links.

Reinforcement learning (RL)-based methods, including multi-agent deep RL, have also been investigated for MCTS. These methods allow UAVs to learn search policies through environmental interaction, typically under a centralized-training and distributed-execution paradigm [31,32,33,34,35]. Although promising in complex scenarios, they require extensive training samples and remain highly sensitive to reward design, which is difficult to calibrate under real-world uncertainty.

Swarm-intelligence and bio-inspired heuristic methods have likewise been extensively studied for MCTS. Methods based on particle swarm optimization, ACO, and related metaheuristics can generate near-optimal paths, but they usually require global environmental information and substantial offline computation, which limits real-time responsiveness [36,37,38,39,40,41,42,43]. Although effective in many settings, these approaches often assume statistically independent target locations and therefore do not exploit spatial-correlation priors that can accelerate target discovery. They also rarely model the closed-loop coupling between sensing quality and motion planning, despite the fact that detection reliability depends on the UAV–target distance and should influence the planned trajectory. Table 1 summarizes how representative methods in these categories differ along the five design dimensions that the present framework addresses jointly.

Despite these advances, existing methods still exhibit three limitations. First, many existing studies treat target states as statistically independent and neglect spatial clustering or regular spacing, both of which are common in realistic deployments. This simplification prevents a search policy from using positive and negative detection events to infer likely target locations in spatially related cells. Second, many approaches rely on centralized controllers or global knowledge of the environment and team state. Such architectures are difficult to reconcile with practical multi-UAV systems, where each platform must make decisions from local and communicated information, and they are vulnerable to scalability limits, single-point failures, and communication delays. Third, conventional search strategies often couple detection and motion planning very tightly, so that each detection action may trigger immediate trajectory replanning. Since target sensing is typically much faster than UAV motion, such coupling introduces unnecessary computation and short-sighted trajectory adjustments. Moreover, most existing methods do not provide a dynamic negotiation-based region partition for resolving inter-UAV conflicts during motion planning. They either rely on static prepartitioning that cannot adapt to evolving belief maps or ignore coverage redundancy and allow multiple UAVs to repeatedly search the same areas.

Accordingly, the core research objective is to design a fully distributed policy for *K* UAVs that, using only locally sensed and neighbor-received information, detects all stationary targets while limiting travel cost, redundant inspection, and application-layer search payload without a central online planner.

To address this objective, we propose a distributed event-driven search framework that integrates pairwise Bayesian inference, positive-event DSF, and decoupled detection–motion planning with dynamic regions. These components form a closed “sense–communicate–act” loop, where sensing (local detection and belief update), communication (DSF), and actuation (waypoint selection and motion) are decoupled in time. The Bayesian model transforms raw sensing outcomes into posterior beliefs, while the DSF algorithm aligns detection evidence across the team through sparse event dissemination. Finally, the decoupled planner converts the maintained beliefs into coordinated detection and motion-planning actions.

The main contributions are summarized as follows.

1. **Spatially correlated online Bayesian belief update.** We model the target location as pairwise target dependence through a distance-dependent interaction kernel and derive a detector from Neyman–Pearson detection theory. These two ingredients yield an online Bayesian belief inference method that explicitly couples spatial inference with UAV detection geometry. Moreover, we prove that its posterior belief is order-invariant, meaning that it depends only on the accumulated detection-event set and not on the order in which local events and received messages are processed.
2. **Event-driven distributed information fusion.** To avoid the communication burden of exchanging full belief maps, we develop a Distributed Selective Flooding (DSF) algorithm that propagates only positive detection events through neighbor-to-neighbor communication. For a fixed connected flooding session with successful delivery and at least the graph-diameter number of rounds, the algorithm achieves finite-time set consensus of positive events under repeated local exchanges, which provides a flexible mechanism for fusing local beliefs while preserving the order-invariance property of the Bayesian update.
3. **Decoupled detection–motion planning with dynamic region adaptation.** We develop a two-phase search policy that separates fast local detection from slower motion replanning. During the detection phase, each UAV greedily orders cells within its field of view by the product of their belief score and the distance-dependent detection probability, so that both target likelihood and sensing reliability influence the schedule. During the motion-planning phase, it selects a heading and waypoint by maximizing a surrogate detection probability, which directly captures the chance of at least one true positive detection in the next stationary sensing phase, rather than summing belief scores. A peer-to-peer responsibility-region adaptation rule transfers high-belief boundary cells between neighboring UAVs only after the requesting UAV has depleted its local high-value cells and has both available neighbors and a nonempty boundary request set, thereby maintaining conflict-free coverage and reducing redundant detection without centralized assignment.

The relationships among the three contributions are summarized as follows. The order-invariance of the Bayesian method in Contribution 1 ensures that posterior beliefs depend on accumulated detection events rather than on their processing order, which justifies the event-set consensus objective of the DSF algorithm in Contribution 2. Once UAVs acquire the same positive-event set, their local belief maps can be aligned without exchanging full probability distributions. The belief maps maintained through DSF then provide the quantitative basis for the belief–detection-probability-ordered greedy detection and the product-form waypoint selection in Contribution 3. Therefore, the three contributions form a self-reinforcing loop in which the detection, communication, and planning mutually support one another.

The rest of the paper is organized as follows. Section 2 formulates the search problem and introduces the target, sensing, communication, and information models. Building on these models, Section 3 develops the Bayesian belief-map update under the Neyman–Pearson detector. To share the resulting detection evidence efficiently, Section 4 presents the conditional DSF positive-event consensus algorithm. Section 5 then introduces the decoupled detection–motion planning strategy and the dynamic region-adaptation mechanism. Furthermore, Section 6 integrates these components into the overall framework and states its conditional properties and computational complexity. Finally, Section 7 reports the numerical experiments and Section 8 concludes the paper.

## 2. Problem Formulation

This section formulates the online cooperative search problem and introduces the target, UAV, communication, and information models used throughout the paper.

### 2.1. Overall Cooperative Search Scenario

**Figure 1:**
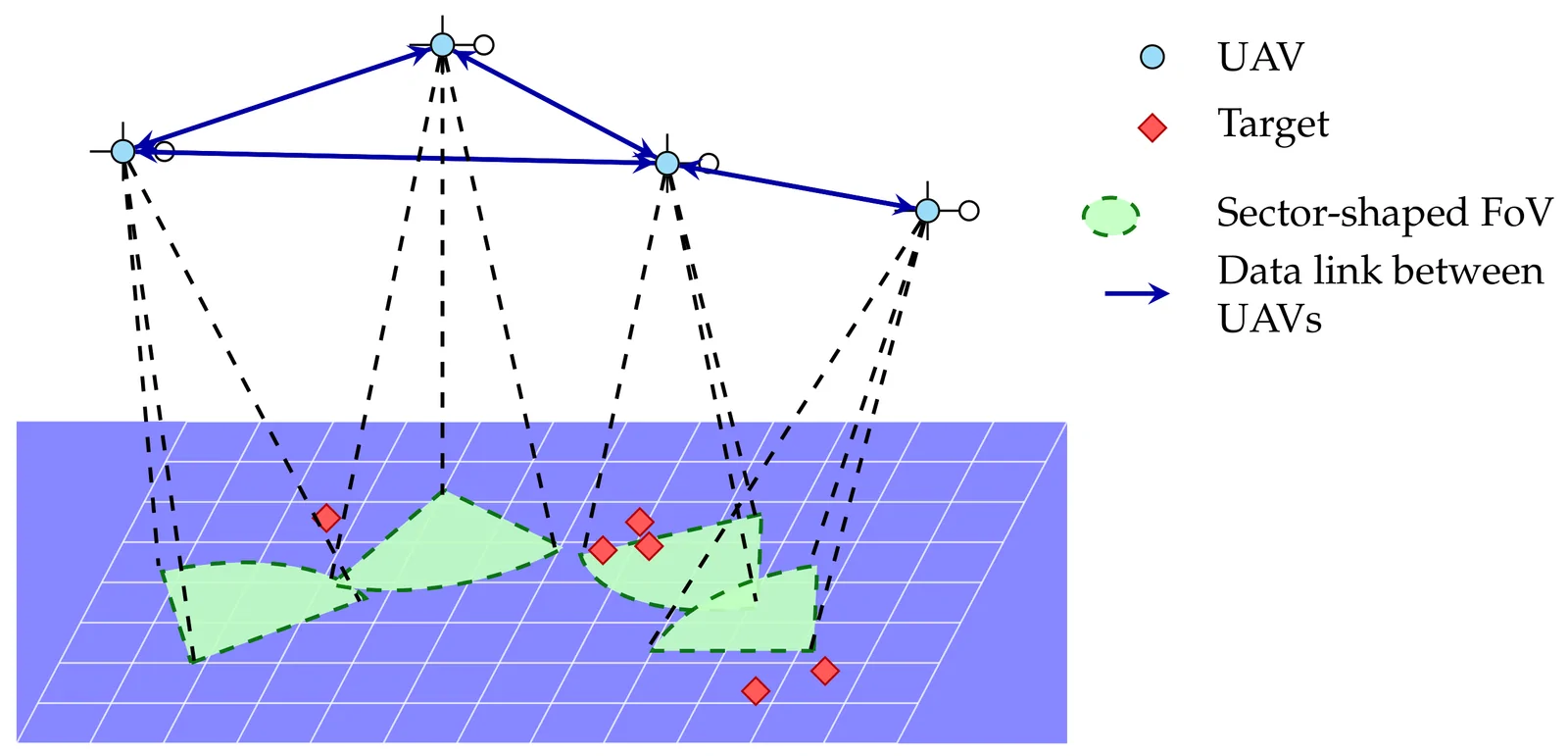
Schematic illustration of the cooperative search scenario. Multiple UAVs search a discretized ground region with sector-shaped FoV, exchange positive detection events through inter-UAV data links, and infer spatially correlated targets without a central coordinator.

Consider a team of UAVs that cooperatively detects an unknown number of stationary targets distributed over a ground region. The prior may include an estimate of their expected number, but the search policy does not observe the target locations. The region is discretized into a two-dimensional grid

(1) $$\begin{array}{c} G=\{(i,j)|i=1,\ldots ,M,\;j=1,\ldots ,N\}, \end{array}$$

where each cell (i,j)∈G has center coordinate $c_{ij}=\left(x_{i},y_{j}\right)\in R^{2}$. The cooperative search scenario over G is illustrated in Figure 1. Each UAV carries a downward-looking sensor with a sector-shaped field of view (FoV). Although the FoV may cover multiple grid cells, the sensor detects one cell at a time and returns a binary observation before switching to another cell. The UAVs exchange detection information through a wireless peer-to-peer network. The targets are not assumed to be independently distributed, and their locations may exhibit spatial correlation patterns such as clustering or structured spacing.

Building on the research objective and contributions stated in Section 1, the core research problem in this paper is to coordinate, without a central planner, how each UAV uses locally acquired and neighbor-received information to exploit spatial correlation among stationary targets, exchange only detection-relevant evidence together with the compact messages required for responsibility negotiation, and schedule detection and motion actions so as to reduce the grid fraction needed for target discovery, repeated coverage, and travel cost. The three limitations identified in the Introduction translate directly into three challenges that any distributed policy must address. First, targets may be spatially correlated. A detection or non-detection at one cell should propagate to related cells through the belief update, but most existing methods assume independent cells and therefore cannot exploit this structure. Second, exchanging full belief maps is bandwidth-prohibitive, yet each of the UAV’s decisions depends on evidence acquired by other team members. Third, detection actions are orders of magnitude faster than UAV motion, so coupling every sensing action to trajectory replanning is inefficient; and without a mechanism to negotiate search regions, multiple UAVs may repeatedly inspect the same areas.

**Figure 2:**
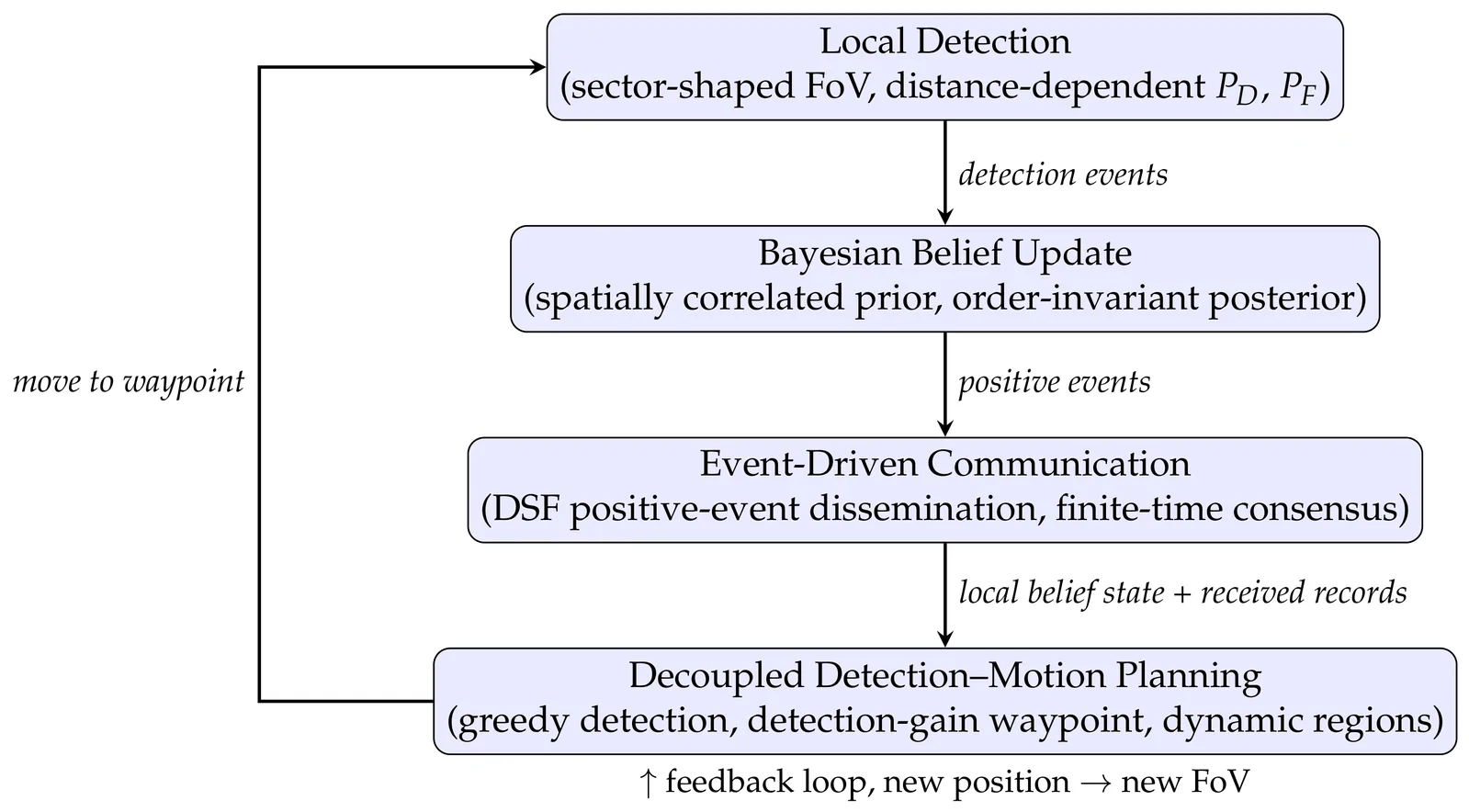
Closed-loop process of the cooperative search scenario, in which local detection, Bayesian belief updating, event-driven communication, and decoupled detection–motion planning form a distributed sense–communicate–act pipeline. The downward arrows denote local detection records, positive-event records, and the updated local score state with received records, respectively, and the return arrow represents the feedback loop in which waypoint execution determines the next detection position and FoV and restarts local detection.

The search process follows the closed loop shown in the flow chart of Figure 2. At each time step, a UAV first performs local detection within its current FoV and obtains binary observations governed by a distance-dependent sensing model. These observations are then incorporated through a Bayesian belief update, which recursively computes posterior target-existence probabilities while accounting for spatial correlations among cells. To share meaningful evidence without exchanging full belief maps, the event-driven communication stage selectively sends positive detection events to neighboring UAVs through a distributed selective flooding algorithm. Finally, the decoupled detection–motion planning stage determines whether the UAV should continue local detection or move to a new position. Motion-planning decisions maximize expected detection gain over dynamically adjusted responsibility regions while penalizing travel cost and suppressing redundant coverage. This decoupled design separates fast local detection from slower motion replanning and delegates inter-UAV coordination to local event-triggered region adaptation, thereby enabling fully distributed implementation.

### 2.2. Spatially Correlated Target Model

The unknown target field is modeled as a binary random field ${\left\{X_{i,j}\right\}}_{(i,j)\in G}$, where $X_{i,j}=1$ indicates that cell (i,j)∈G contains a stationary target, and $X_{i,j}=0$ otherwise. Let $p_{i,j}^{\left(0\right)}\in \left[0,1\right]$ denote the spatially varying prior probability. Separately, if *s* independent uniform target placements over the MN cells are used as a reference model, define the prior probability as follows

$$P\left(X_{i,j}=1\right)=p^{\left(0\right)}=1-{\left(1-1/NM\right)}^{s}.$$

The spatial target model is intended not merely to assign independent priors to cells, but to characterize how the state of one cell probabilistically affects the state of another. Concrete examples of spatially correlated stationary targets include vehicles parked in convoy or formation areas, survivors concentrated near shelters after a disaster, and sensor nodes deployed at approximately regular spacing. In each case, a detection or nondetection at one location changes the plausibility of targets at spatially related locations—for instance, finding one vehicle in a convoy makes adjacent cells more likely to contain additional vehicles, while a thorough sweep of an empty stretch of road suppresses the expected count along that corridor. We model these pairwise probabilistic relations through distance-dependent conditional probabilities.

For two cells (i,j) and (n,m), define their intercell distance as

$$d_{ij,nm}={∥c_{ij}-c_{nm}∥}_{2}.$$

The spatial interaction strength is described by the Gaussian kernel

$$\kappa \left(d\right)=\eta \exp\;\left(-\frac{{\left(d-r\right)}^{2}}{2\sigma^{2}}\right),$$

where η>0 controls the maximum coupling strength, r>0 denotes the characteristic spacing at which two target locations are most strongly correlated, and σ>0 determines the effective spatial range of the interaction. Cells whose separation is close to *r* exert the strongest probabilistic influence, while cells that are much closer or farther away have weaker influence.

Using $\kappa (d_{ij,nm})$, we define the conditional probability that cell (i,j) contains a target, given that cell (n,m) contains a target, as

(2) $$\begin{array}{c} P\left(X_{i,j}=1|X_{n,m}=1\right)=\left\{\begin{array}{cc} 1, & (i,j)=(n,m),\\ \frac{p^{\left(0\right)}+\kappa \left(d_{ij,nm}\right)}{1+\kappa (d_{ij,nm})}, & (i,j)\neq (n,m). \end{array}\right. \end{array}$$

Equation (2) increases the target-existence probability of cell (i,j) above the marginal prior when (i,j) is spatially correlated with an occupied cell (n,m). The increment is governed by $\kappa (d_{ij,nm})$, so the effect is strongest near the characteristic spacing *r* and vanishes as the interaction weakens.

Similarly, when cell (n,m) is known to be empty, Bayes’ rule and the symmetry of the pairwise interaction lead to

(3) $$\begin{array}{cc} P(X_{i,j}=1|X_{n,m}=0) & =\frac{P\left(X_{i,j}=1\right)P\left(X_{n,m}=0|X_{i,j}=1\right)}{P(X_{n,m}=0)}\\ \; & =\left\{\begin{array}{cc} 0, & (i,j)=(n,m),\\ \frac{p^{\left(0\right)}}{1+\kappa (d_{ij,nm})}, & (i,j)\neq (n,m). \end{array}\right. \end{array}$$

**Figure 3:**
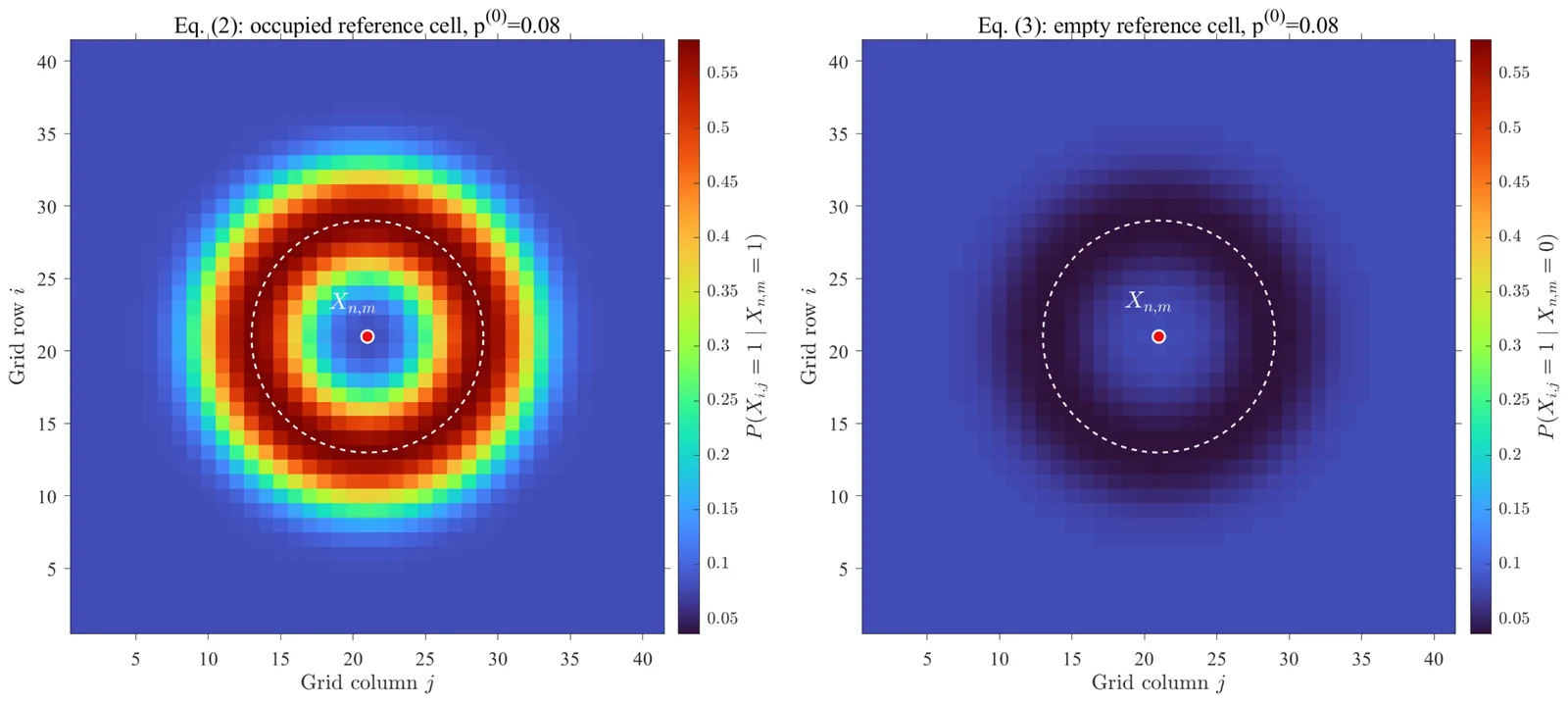
Heatmap visualization of the spatially correlated target model with parameters η=1.20, r=8.0, and σ=2.2. The (**left**) panel shows $P(X_{i,j}=1|X_{n,m}=1)$ in (2), where an occupied reference cell raises the target-existence probability of cells whose distance is close to the characteristic spacing *r*. The (**right**) panel shows $P(X_{i,j}=1|X_{n,m}=0)$ in (3), where an empty reference cell suppresses the probability of the same spatially correlated cells. The dashed ring indicates the strongest interaction region induced by $\kappa (d_{ij,nm})$.

Equation (3) decreases the target-existence probability of cell (i,j) when a strongly related cell (n,m) is known to be empty. Equations (2) and (3) therefore provide a compact pairwise conditional model for spatially correlated target locations. The resulting conditional probability fields under the two reference-cell states are visualized in Figure 3.

This formulation is directly used by the Bayesian update in the subsequent sections. A positive detection at one cell propagates probability mass to cells with large κ(d), while a negative detection suppresses the belief of strongly related cells. Consequently, the search policy can exploit target-location structure rather than treating all grid cells as independent candidates.

### 2.3. UAV Model

The search task is performed by *K* UAVs flying at a fixed altitude. Since the altitude remains constant during the mission, each UAV is modeled as a planar point mass. Let

$$q_{k}\left(t\right)={\left[x_{k}\left(t\right),y_{k}\left(t\right)\right]}^{⊤}\in R^{2},\;\psi_{k}\left(t\right)\in \left[0,2\pi \right)$$

denote the planar position and heading angle of UAV *k* at time *t*, respectively. The corresponding unit heading vector is

$$g_{k}\left(t\right)=g\left(\psi_{k}\left(t\right)\right)={\left[\cos\psi_{k}\left(t\right),\sin\psi_{k}\left(t\right)\right]}^{⊤}\in R^{2}.$$

With bounded cruising speed $v_{k}\left(t\right)$, the planar kinematics is written as

$$q_{k}\left(t+\Delta t\right)=q_{k}\left(t\right)+v_{k}\left(t\right)\Delta t\;g_{k}\left(t\right),$$

where $0\leq v_{k}\left(t\right)\leq v_{\max}$. Hence, the search trajectory is determined by the planar position, heading, and admissible speed profile.

For cooperative search, each UAV is assigned a time-varying *responsibility region.* Let

(4) $${\left\{R_{k}\left(t\right)\right\}}_{k=1}^{K},\;R_{k}\left(t\right)\subseteq G,$$

denote a collection of pairwise disjoint grid subsets, where UAV *k* is primarily responsible for detection and motion-planning decisions inside $R_{k}\left(t\right)$. At the beginning of the mission, these regions are generated from the initial UAV positions according to the Voronoi rule

(5) $$R_{k}\left(0\right)=V_{k}=\left\{\left(i,j\right)\in G|∥c_{ij}-q_{k}^{\left(0\right)}{∥}_{2}\leq {∥c_{ij}-q_{\ell }^{\left(0\right)}∥}_{2},\;\forall \ell \neq k\right\},$$

which assigns each grid cell to its nearest initial UAV and yields $R_{k}\left(0\right)\cap R_{\ell }\left(0\right)=\emptyset$ for k≠ℓ. These regions provide an initial conflict-free partition of the search area, while their dynamic adaptation is developed later in the motion-planning module.

A UAV at position $q\in R^{2}$ with heading angle ψ observes the environment through a sector-shaped FoV. Define

$$r_{ij}\left(q\right)=c_{ij}-q,\;g\left(\psi \right)={\left[\cos\psi ,\sin\psi \right]}^{⊤}.$$

The cells covered by the FoV are

(6) $$S\left(q,\psi \right)=\left\{\left(i,j\right)\in G\;|\;{∥r_{ij}\left(q\right)∥}_{2}\leq R_{\max},\;r_{ij}{\left(q\right)}^{⊤}g\left(\psi \right)\geq {∥r_{ij}\left(q\right)∥}_{2}\cos\left(\theta /2\right)\right\},$$

where $R_{\max}$ is the detection radius, and θ is the angular aperture. The second inequality in (6) is the inner-product form of the angular constraint. At time *t*, the visible cell set of UAV *k* is

$$F_{k}\left(t\right)=S\left(q_{k}\left(t\right),\psi_{k}\left(t\right)\right).$$

**Figure 4:**
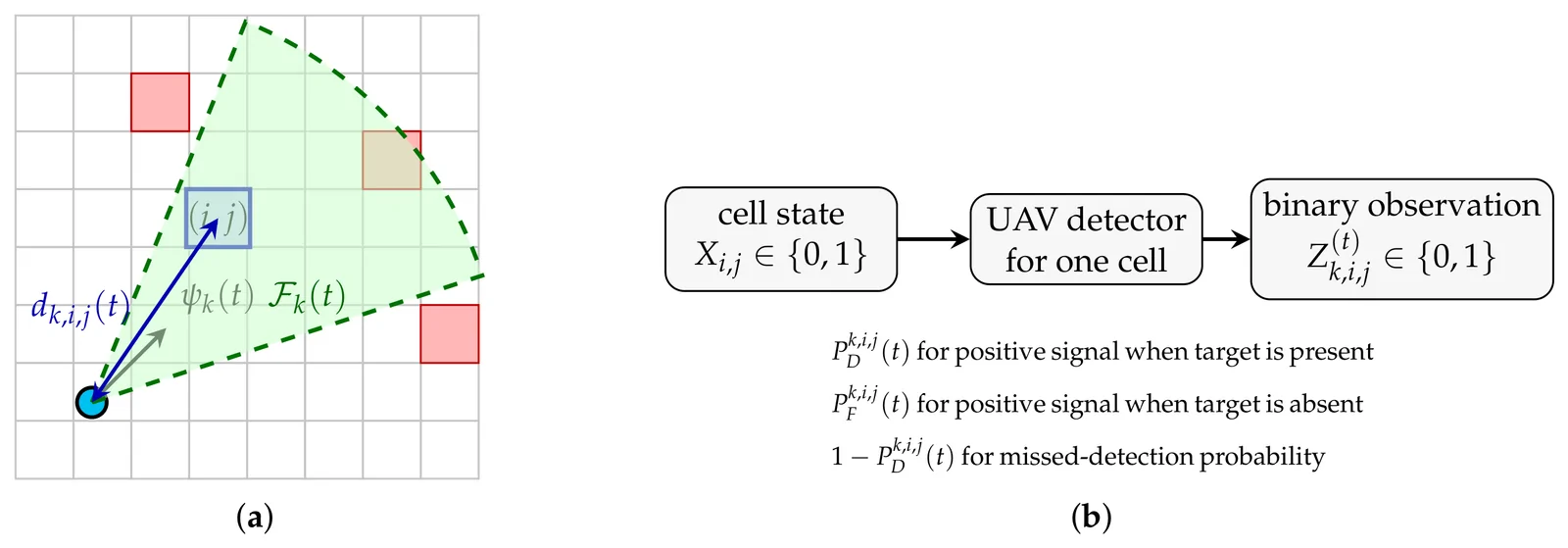
UAV sensing geometry and binary observation model. In (**a**), the red cells illustrate target-occupied cells, the translucent green sector is the visible set $F_{k}\left(t\right)$, its dashed green outline marks the FoV boundary, the blue cell is the cell selected for interrogation, and the blue segment indicates its distance from UAV *k*. At each sensing action, UAV *k* interrogates one visible cell and obtains the binary observation in (**b**), whose reliability is characterized by the detection, false-alarm, and missed-detection probabilities.

Each UAV is equipped with an onboard detector that interrogates one visible grid cell at a time, as illustrated in Figure 4. When UAV *k* detects cell (i,j) at time *t*, it obtains a binary observation $Z_{k,i,j}^{\left(t\right)}\in \left\{0,1\right\}$, where $Z_{k,i,j}^{\left(t\right)}=1$ denotes a positive detection signal, and $Z_{k,i,j}^{\left(t\right)}=0$ denotes the absence of a positive signal. Since onboard sensing is imperfect, two reliability quantities are associated with this observation. The first is the detection probability

(7) $$\begin{array}{c} P_{D}^{k,i,j}\left(t\right)=P\left(Z_{k,i,j}^{\left(t\right)}=1|X_{i,j}=1\right), \end{array}$$

which characterizes the probability of reporting a positive signal when the target is present. The second is the false-alarm probability

(8) $$\begin{array}{c} P_{F}^{k,i,j}\left(t\right)=P\left(Z_{k,i,j}^{\left(t\right)}=1|X_{i,j}=0\right) \end{array}$$

which characterizes the probability of reporting a positive signal when the target is absent. Equivalently, $1-P_{D}^{k,i,j}\left(t\right)$ is the missed-detection probability. Physically meaningful distance-dependent expressions for $P_{D}^{k,i,j}\left(t\right)$ and $P_{F}^{k,i,j}\left(t\right)$, with $d_{k,i,j}\left(t\right)={∥q_{k}\left(t\right)-c_{ij}∥}_{2}$, are derived under a Neyman–Pearson model in Section 3.1.

### 2.4. Communication Network and Event-Driven Triggering

At each time *t*, the inter-UAV connectivity is modeled by an undirected graph $G_{c}\left(t\right)=\left(U,E\left(t\right)\right)$, where U={1,…,K} is the UAV set, and E(t) is the set of available communication links. An edge (k,ℓ)∈E(t) exists if and only if the Euclidean distance between UAVs *k* and *ℓ* does not exceed the prescribed communication radius $d_{\mathrm{com}}$.

(9) $$E\left(t\right)=\left\{\left(k,\ell \right)\in U\times U|\;∥q_{k}\left(t\right)-q_{\ell }{\left(t\right)∥}_{2}\leq d_{\mathrm{com}},\;k\neq \ell \right\},$$

where $q_{k}\left(t\right)$ denotes the planar position of UAV *k*. Accordingly, the neighbor set of UAV *k* is defined as

(10) $$N_{k}\left(t\right)=\left\{\ell \in U\setminus \left\{k\right\}|\;∥q_{k}\left(t\right)-q_{\ell }{\left(t\right)∥}_{2}\leq d_{\mathrm{com}}\right\}.$$

Since the UAVs move during the mission, $G_{c}\left(t\right)$ is generally time-varying. Communication can occur only along the available edges of $G_{c}\left(t\right)$, and information can propagate through the network in a multi-hop manner whenever the instantaneous graph is not fully disconnected.

Although $G_{c}\left(t\right)$ specifies which UAV pairs can communicate at time *t*, it does not require communication at every time step. In practical multi-UAV systems, frequent communication repeatedly activates wireless radios, increases energy consumption, and consumes limited onboard bandwidth [26]. Moreover, exchanging full belief maps of size |G| at every time step is unnecessary when most observations are routine negative detections. The proposed framework therefore uses event-driven communication over the peer-to-peer network.

The event-driven mechanism is used in two distinct parts of the framework. The first is DSF-based information consensus. From the local viewpoint of UAV *k*, DSF communication is activated only when UAV *k* holds positive-event innovations that have not yet been sent to its current neighbors. These innovations may be generated by its own detector or received from other UAVs in earlier exchanges. This trigger prevents routine negative detections from being flooded through the network. The detailed DSF triggering rule is given in Section 4.

The second is coordination for responsibility-region adaptation. This trigger is used during the motion-planning stage. UAV *k* compares the high-value belief mass and cell count remaining in its own region with the requestable high-value mass and cell count in neighboring regions. The workload-balancing gate suppresses negotiation while the local remainder still substantially exceeds what could be gained from a neighbor, thereby reducing frequent low-benefit boundary changes, as detailed in Section 5.2.2.

In this way, the graph $G_{c}\left(t\right)$ provides the physical peer-to-peer communication links, while the event-driven rules determine whether these links are used for positive-event consensus or boundary-cell negotiation.

### 2.5. Information Structure and Belief State

Each UAV *k* maintains a local information set that accumulates detection evidence available up to time *t*. This evidence has two sources, namely onboard detection events and messages received from neighboring UAVs.

When UAV *k* detects cell (n,m) at time τ, it obtains a binary observation $Z_{k,n,m}^{\left(\tau \right)}\in \left\{0,1\right\}$ together with the associated detection probability $P_{D}^{k,n,m}\left(\tau \right)$ and false-alarm probability $P_{F}^{k,n,m}\left(\tau \right)$, which are determined by the distance-dependent detection model (14)–(15). The set of local detection records collected up to time *t* is denoted by

(11) $$Z_{k}^{\left(t\right)}=\left\{(n,m,\;Z_{k,n,m}^{\left(\tau \right)},\;P_{D}^{k,n,m}\left(\tau \right),\;P_{F}^{k,n,m}\left(\tau \right))|\tau \leq t\right\}.$$

Each tuple records the cell location, the measured binary outcome, and the detection parameters that characterize sensing reliability.

The UAVs share detection outcomes over the communication network defined in Section 2.4. Let $N_{k}\left(t\right)$ be the neighbor set of UAV *k* at time *t*, as given in (10). The set of positive detection events received from neighbors up to time *t* is

(12) $$E_{k}^{\left(t\right)}=\left\{\left(n,m,\;Z_{\ell ,n,m}^{\left(\tau \right)},\;P_{D}^{\ell ,n,m}\left(\tau \right),\;P_{F}^{\ell ,n,m}\left(\tau \right)\right)|\ell \in N_{k}\left(\tau \right),\;\tau \leq t\right\}.$$

The total information set available to UAV *k* at time *t* is then the union of its own detection records and the events received from neighbors.

(13) $$I_{k}^{\left(t\right)}=Z_{k}^{\left(t\right)}\cup E_{k}^{\left(t\right)}.$$

Based on $I_{k}^{\left(t\right)}$, UAV *k* recursively computes a local belief map

$$p_{k}^{\left(t\right)}={\left\{p_{k,i,j}^{\left(t\right)}\right\}}_{(i,j)\in G},\;$$

where $p_{k,i,j}^{\left(t\right)}$ is the normalized Bayesian belief value produced by the pairwise likelihood update. As shown in the next section, its value is updated recursively through Bayesian rules. It equals the exact posterior marginal $P(X_{i,j}=1|I_{k}^{\left(t\right)})$ when the initial prior and pairwise conditionals are compatible with an underlying joint model, the event likelihoods are correctly specified, and the accumulated events are conditionally independent given $X_{i,j}$. When these conditions do not hold, it is a normalized pairwise Bayesian surrogate rather than a calibrated posterior probability. The experimental maps are interpreted as Bayesian belief maps subject to this qualification.

## 3. Bayesian Belief Map Update During Search

The local belief map $p_{k}^{\left(t\right)}$ represents UAV *k*’s current probabilistic knowledge of the target field. It supports two online decisions, namely selecting the next cell to detect within the current FoV and determining where to move in order to cover remaining high-probability areas efficiently. Maintaining accurate and mutually consistent belief maps is therefore essential for coordinated search.

This section first derives the distance-dependent detection probabilities using the Neyman–Pearson framework and then develops a recursive Bayesian update rule that allows each UAV to incorporate new evidence into its belief map in a computationally efficient and order-invariant manner.

### 3.1. Neyman–Pearson Detection Model

This subsection develops a physically meaningful sensing model for the detection and false-alarm probabilities introduced in Section 2.3. The objective is to express $P_{D}^{k,i,j}\left(t\right)$ and $P_{F}^{k,i,j}\left(t\right)$ as functions of the distance between UAV *k* and cell (i,j), thereby coupling sensing reliability with UAV position.

Specifically, we adopt a scalar received-signal model that links the sensed amplitude to the target state and the UAV–cell geometry. When UAV *k* detects cell (i,j) at time *t*, the received signal amplitude is modeled as a random variable $Y_{k,i,j}^{\left(t\right)}\in R$. Under the null hypothesis $H_{0}$ for target absence and the alternative hypothesis $H_{1}$ for target presence, the signal distributions are modeled as follows.

$$H_{0}:Y_{k,i,j}^{\left(t\right)}∼N\left(0,\sigma_{n}^{2}\right),\;H_{1}:Y_{k,i,j}^{\left(t\right)}∼N\left(\mu_{k,i,j}\left(t\right),\sigma_{n}^{2}\right),$$

where $\sigma_{n}^{2}$ denotes the detector noise variance. The mean signal amplitude under $H_{1}$ decays with the UAV-to-cell distance $d_{k,i,j}\left(t\right)={∥q_{k}\left(t\right)-c_{ij}∥}_{2}$ according to a free-space path-loss model [44,45].

$$\mu_{k,i,j}\left(t\right)=\frac{\alpha }{d_{k,i,j}{\left(t\right)}^{\gamma }},$$

with signal intensity constant α>0 and path-loss exponent γ≥2.

By the Neyman–Pearson Lemma [46], the uniformly most powerful test under a prescribed false-alarm constraint is the likelihood-ratio test. For the Gaussian model above, the log-likelihood ratio reduces to a linear threshold test on the received signal.

$$Y_{k,i,j}^{\left(t\right)}{≷}_{H_{0}}^{H_{1}}\tau .$$

For a prescribed false alarm probability $P_{F}^{⋆}$, the threshold τ is determined by

$$P_{F}^{⋆}=P\left(Y_{k,i,j}^{\left(t\right)}>\tau |H_{0}\right)=Q\;\left(\frac{\tau }{\sigma_{n}}\right),$$

where $Q\left(x\right)=\frac{1}{\sqrt{2\pi }}\int_{x}^{\infty }e^{-t^{2}/2}\;dt$ is the complementary cumulative distribution function of the standard normal distribution. Hence, the threshold associated with the prescribed false-alarm probability $P_{F}^{⋆}$ is $\tau^{*}=\sigma_{n}\;Q^{-1}\left(P_{F}^{⋆}\right)$. The resulting detection and false-alarm probabilities are

(14) $$\begin{array}{cc} P_{D}^{k,i,j}\left(t\right) & =P\left(Y_{k,i,j}^{\left(t\right)}>\tau^{*}|H_{1}\right)=Q\;\left(Q^{-1}\left(P_{F}^{⋆}\right)-\frac{\alpha }{\sigma_{n}\;d_{k,i,j}{\left(t\right)}^{\gamma }}\right), \end{array}$$

(15) $$\begin{array}{cc} P_{F}^{k,i,j}\left(t\right) & =P\left(Y_{k,i,j}^{\left(t\right)}>\tau^{*}|H_{0}\right)=P_{F}^{⋆}. \end{array}$$

**Figure 5:**
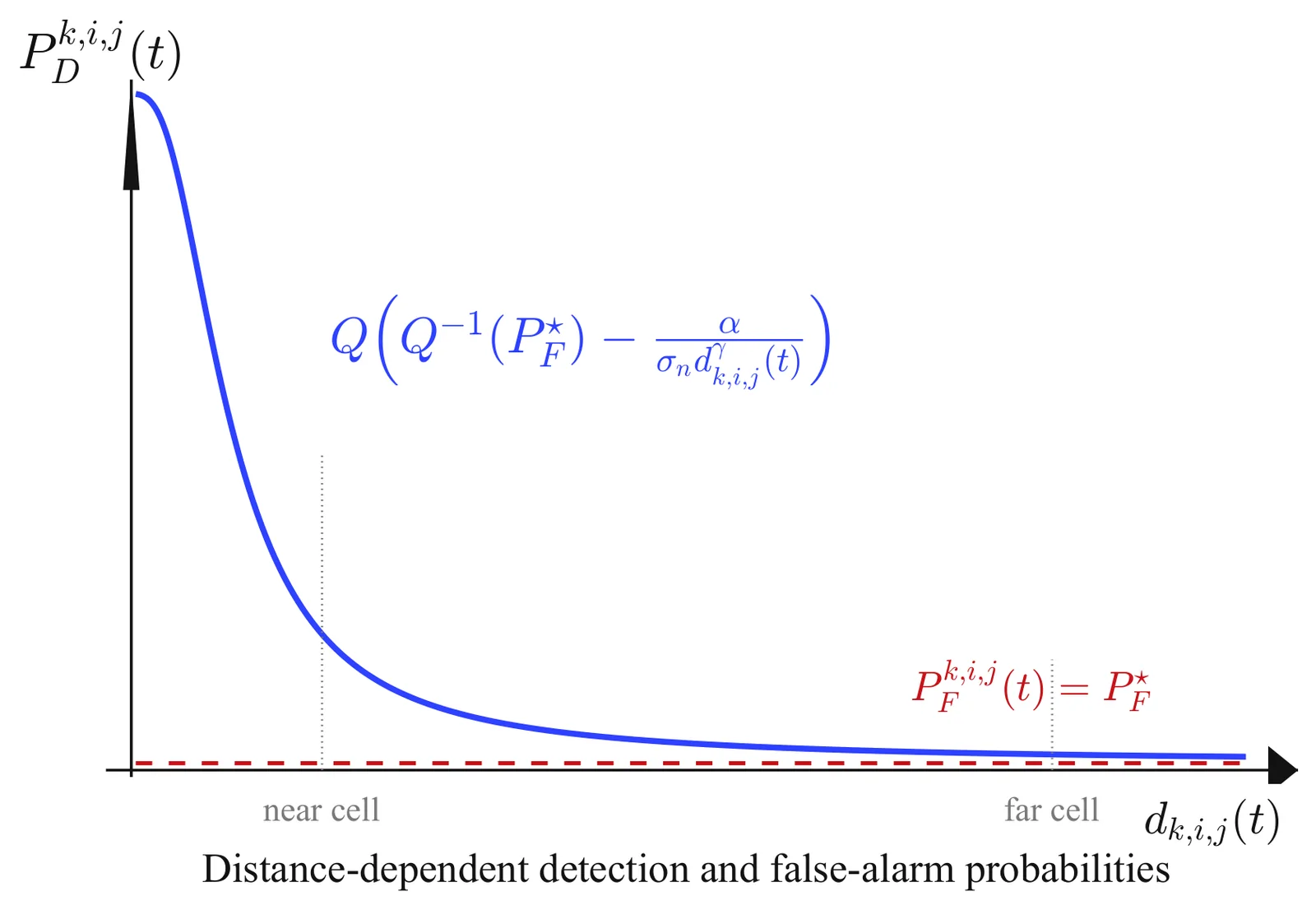
Schematic illustration of the distance-dependent detection probability with parameters α=1.35, γ=1.55, $\sigma_{n}=0.42$ and $P_{F}^{⋆}=0.03$. The blue curve is the detection probability $P_{D}^{k,i,j}\left(t\right)$, which decreases with the effective UAV-to-cell distance $d_{k,i,j}^{\mathrm{eff}}\left(t\right)$. The red dashed line is the constant nominal false-alarm probability $P_{F}^{⋆}$ and the gray dotted vertical lines mark representative distances for the labeled near and far cells.

As illustrated in Figure 5, $P_{D}^{k,i,j}\left(t\right)$ decreases monotonically with the UAV-to-cell distance $d_{k,i,j}\left(t\right)$. The sensing quality is determined by the signal-to-noise ratio ${\mathrm{SNR}}_{k,i,j}\left(t\right)=\alpha /\left(\sigma_{n}d_{k,i,j}{\left(t\right)}^{\gamma }\right)$. As the UAV approaches a grid cell, the SNR and the detection probability both increase. This distance dependence provides a physically meaningful link between sensing reliability and UAV position.

### 3.2. Recursive Bayesian Belief Update

With the distance-dependent probabilities $P_{D}^{k,i,j}\left(t\right)$ and $P_{F}^{k,i,j}\left(t\right)$ in (14) and (15) and the spatial correlation model in (2) and (3), this subsection derives the recursive update rule for the local belief map $p_{k}^{\left(t\right)}$ and establishes its fundamental order-invariance property.

Specifically, suppose that at time *t*, UAV *k* receives a set of newly arrived events

$$\Delta I_{k}^{\left(t\right)}:=I_{k}^{\left(t\right)}\setminus I_{k}^{\left(t-1\right)}.$$

This set contains the incremental information acquired at time *t* through either local detection or received messages. Each element $e\in \Delta I_{k}^{\left(t\right)}$ has the form

$$e=(n,m,z,P_{D},P_{F}),$$

where (n,m)∈G is the grid index, z∈{0,1} is the binary detection outcome of cell (n,m), and $\left(P_{D},P_{F}\right)$ denotes the corresponding sensing-reliability parameters. The purpose of the update is to incorporate this incremental information into the local belief map $p_{k}^{\left(t\right)}$.

When new events in $\Delta I_{k}^{\left(t\right)}$ arrive, UAV *k* updates its belief map recursively according to Algorithm 1.
**Algorithm 1** Bayesian Local Belief Update—From the Viewpoint of UAV *k***Require:** local belief map $p_{k}^{\left(t-1\right)}$, incremental information set $\Delta I_{k}^{\left(t\right)}$ and its cardinality $M=\left|\Delta I_{k}^{\left(t\right)}\right|$.**Ensure:** local belief map $p_{k}^{\left(t\right)}$.**Initialize:** $u^{\left(0\right)}=p_{k}^{\left(t-1\right)}$ (record sent/received history for each neighbor).**for** r=1,2,…,M **do**    Select element $e_{r}=\left(n_{r},m_{r},z_{r},P_{D}^{\left(r\right)},P_{F}^{\left(r\right)}\right)\in \Delta I_{k}^{\left(t\right)}$;    **for** (i,j)∈G **do**        Compute $L_{n_{r},m_{r},i,j}^{\left(1\right)}=P\left(X_{n_{r},m_{r}}=1|X_{i,j}=1\right)$ by (2) and $L_{n_{r},m_{r},i,j}^{\left(0\right)}=P\left(X_{n_{r},m_{r}}=1|X_{i,j}=0\right)$ by (3).        Compute(16a)$$\begin{array}{cc} D_{n_{r},m_{r},i,j}^{\left(1\right)} & =L_{n_{r},m_{r},i,j}^{\left(1\right)}P_{D}^{\left(r\right)}+\left(1-L_{n_{r},m_{r},i,j}^{\left(1\right)}\right)P_{F}^{\left(r\right)}, \end{array}$$(16b)$$\begin{array}{cc} D_{n_{r},m_{r},i,j}^{\left(0\right)} & =L_{n_{r},m_{r},i,j}^{\left(0\right)}P_{D}^{\left(r\right)}+\left(1-L_{n_{r},m_{r},i,j}^{\left(0\right)}\right)P_{F}^{\left(r\right)}, \end{array}$$        If $z_{r}=1$, update$$u_{i,j}^{\left(r\right)}=\frac{D_{n_{r},m_{r},i,j}^{\left(1\right)}\;u_{i,j}^{\left(r-1\right)}}{D_{n_{r},m_{r},i,j}^{\left(1\right)}\;u_{i,j}^{\left(r-1\right)}+D_{n_{r},m_{r},i,j}^{\left(0\right)}\;\left(1-u_{i,j}^{\left(r-1\right)}\right)},$$
otherwise, update$$u_{i,j}^{\left(r\right)}=\frac{\left(1-D_{n_{r},m_{r},i,j}^{\left(1\right)}\right)\;u_{i,j}^{\left(r-1\right)}}{\left(1-D_{n_{r},m_{r},i,j}^{\left(1\right)}\right)\;u_{i,j}^{\left(r-1\right)}+\left(1-D_{n_{r},m_{r},i,j}^{\left(0\right)}\right)\;\left(1-u_{i,j}^{\left(r-1\right)}\right)}.$$    **end for****end for**Set $p_{k}^{\left(t\right)}=u^{\left(M\right)}.$

Since multiple events may arrive sequentially, it is important to determine whether the final belief depends on the order in which they are processed. The following theorem establishes the order-invariance of the recursive update.

**Theorem** **1** (Order-Invariance of Event-Driven Bayesian Updates)**.** *Consider the incremental information set $\Delta I_{k}^{\left(t\right)}=\left\{e_{1},\ldots ,e_{M}\right\}$, where each event is $e_{r}=\left(n_{r},m_{r},z_{r},P_{D}^{\left(r\right)},P_{F}^{\left(r\right)}\right)$. Suppose the pairwise likelihood factors $P(e_{r}|X_{i,j}=s)$ for s∈{0,1} are computed once from the pre-update belief map and treated as constants throughout the batch update. Then, the output $p_{k}^{\left(t\right)}$ of Algorithm 1 is independent of the processing order of ${\left\{e_{r}\right\}}_{r=1}^{M}$. Moreover, for all (i,j)∈G,*

(17) $$p_{k,i,j}^{\left(t\right)}=\frac{p_{k,i,j}^{\left(t-1\right)}\prod_{r=1}^{M}P\left(e_{r}|X_{i,j}=1\right)}{p_{k,i,j}^{\left(t-1\right)}\prod_{r=1}^{M}P\left(e_{r}|X_{i,j}=1\right)+\left(1-p_{k,i,j}^{\left(t-1\right)}\right)\prod_{r=1}^{M}P\left(e_{r}|X_{i,j}=0\right)},$$

*where*

(18) $$\begin{array}{c} P\left(e_{r}|X_{i,j}=s\right)=P\left(Z_{n_{r},m_{r}}=z_{r}|X_{i,j}=s\right),\forall s\in \left\{0,1\right\}, \end{array}$$

*denotes the conditional probability that cell $\left(n_{r},m_{r}\right)$ generates the binary outcome $z_{r}$, given the grid state $X_{i,j}=s$ and the detection parameters $P_{D}^{\left(r\right)}$ and $P_{F}^{\left(r\right)}$.*

**Proof.**  The proof proceeds in two steps. First, we show that each recursive update in Algorithm 1 is equivalent to a Bayesian update with a single detection event. Second, we show that repeated application yields a product-form posterior that is invariant to event ordering.

Fix a grid cell (i,j)∈G and consider the *r*-th event $e_{r}=\left(n_{r},m_{r},z_{r},P_{D}^{\left(r\right)},P_{F}^{\left(r\right)}\right)$. It follows from Bayes’ theorem that

(19) $$\begin{array}{cc} P(Z_{n_{r},m_{r}}=z_{r}|X_{i,j}=s)= & P(Z_{n_{r},m_{r}}=z_{r},X_{n_{r},m_{r}}=1|X_{i,j}=s)\\ \; & +P(Z_{n_{r},m_{r}}=z_{r},X_{n_{r},m_{r}}=0|X_{i,j}=s)\\ = & P\left(X_{n_{r},m_{r}}=1|X_{i,j}=s\right)P\left(Z_{n_{r},m_{r}}=z_{r}|X_{n_{r},m_{r}}=1\right)\\ \; & +P\left(X_{n_{r},m_{r}}=0|X_{i,j}=s\right)P\left(Z_{n_{r},m_{r}}=z_{r}|X_{n_{r},m_{r}}=0\right). \end{array}$$

Using (18) and the quantities $D_{n_{r},m_{r},i,j}^{\left(1\right)}$ and $D_{n_{r},m_{r},i,j}^{\left(0\right)}$ defined in (16a) and (16b), we obtain

(20) $$\begin{array}{c} P\left(e_{r}|X_{i,j}=s\right)=\left\{\begin{array}{cc} D_{n_{r},m_{r},i,j}^{\left(s\right)}, & \mathrm{if}\;z_{r}=1,\\ 1-D_{n_{r},m_{r},i,j}^{\left(s\right)}, & \mathrm{if}\;z_{r}=0, \end{array},\forall s\in \left\{0,1\right\}.\right. \end{array}$$

Since $u_{i,j}^{\left(0\right)}=p_{k,i,j}^{\left(t-1\right)}=P\left(X_{i,j}=1|I_{k}^{\left(t-1\right)}\right)$, Bayes’ theorem gives the following posterior after incorporating $e_{1}$, i.e.,

(21) $$P\left(X_{i,j}=1|I_{k}^{\left(t-1\right)}\cup \left\{e_{1}\right\}\right)=\frac{P\left(e_{1}|X_{i,j}=1\right)\;u_{i,j}^{\left(0\right)}}{P\left(e_{1}|X_{i,j}=1\right)\;u_{i,j}^{\left(0\right)}+P\left(e_{1}|X_{i,j}=0\right)\;\left(1-u_{i,j}^{\left(0\right)}\right)},$$

This expression matches the likelihood term used in Algorithm 1. Hence,

$$u_{i,j}^{\left(1\right)}=P\left(X_{i,j}=1|I_{k}^{\left(t-1\right)}\cup \left\{e_{1}\right\}\right).$$

Applying the same argument recursively for r=1,…,M gives

(22) $$\begin{array}{cc} u_{i,j}^{\left(r\right)}= & P\left(X_{i,j}=1|I_{k}^{\left(t-1\right)}\cup \left\{e_{1},\ldots ,e_{r}\right\}\right)\\ = & \frac{P\left(e_{r}|X_{i,j}=1\right)\;u_{i,j}^{\left(r-1\right)}}{P\left(e_{r}|X_{i,j}=1\right)\;u_{i,j}^{\left(r-1\right)}+P\left(e_{r}|X_{i,j}=0\right)\;\left(1-u_{i,j}^{\left(r-1\right)}\right)}, \end{array}$$

Repeated substitution yields

$$u_{i,j}^{\left(M\right)}=\frac{u_{i,j}^{\left(0\right)}\prod_{r=1}^{M}P\left(e_{r}|X_{i,j}=1\right)}{u_{i,j}^{\left(0\right)}\prod_{r=1}^{M}P\left(e_{r}|X_{i,j}=1\right)+\left(1-u_{i,j}^{\left(0\right)}\right)\prod_{r=1}^{M}P\left(e_{r}|X_{i,j}=0\right)}.$$

Since $p_{k,i,j}^{\left(t\right)}=u_{i,j}^{\left(M\right)}=P\left(X_{i,j}=1|I_{k}^{\left(t-1\right)}\cup \Delta I_{k}^{\left(t\right)}\right)$, we obtain

(23) $$p_{k,i,j}^{\left(t\right)}=\frac{p_{k,i,j}^{\left(t-1\right)}\prod_{r=1}^{M}P\left(e_{r}|X_{i,j}=1\right)}{p_{k,i,j}^{\left(t-1\right)}\prod_{r=1}^{M}P\left(e_{r}|X_{i,j}=1\right)+\left(1-p_{k,i,j}^{\left(t-1\right)}\right)\prod_{r=1}^{M}P\left(e_{r}|X_{i,j}=0\right)},$$

which implies that the expression of $p_{k,i,j}^{\left(t\right)}$ in (23) depends on the events ${\left\{e_{r}\right\}}_{r=1}^{M}$ only through the products

$$\prod_{r=1}^{M}P\left(e_{r}|X_{i,j}=s\right),\;s\in \left\{0,1\right\}.$$

Since multiplication is commutative and associative, these products are independent of the event order. The final posterior $p_{k,i,j}^{\left(t\right)}$ is therefore invariant to any permutation of ${\left\{e_{r}\right\}}_{r=1}^{M}$, which completes the proof.    ☐

**Remark** **1.** *Although the local belief map is updated sequentially, the resulting posterior remains order-independent. Consequently, the posterior admits the following product-form representation.*

(24) $$p_{k,i,j}^{\left(t\right)}=\frac{p_{k,i,j}^{\left(0\right)}\prod_{e_{r}\in I_{k}^{\left(t\right)}}P\left(e_{r}|X_{i,j}=1\right)}{p_{k,i,j}^{\left(0\right)}\prod_{e_{r}\in I_{k}^{\left(t\right)}}P\left(e_{r}|X_{i,j}=1\right)+\left(1-p_{k,i,j}^{\left(0\right)}\right)\prod_{e_{r}\in I_{k}^{\left(t\right)}}P\left(e_{r}|X_{i,j}=0\right)},\;\forall \left(i,j\right)\in G.$$

*Consider two UAVs k and ℓ initialized with identical belief maps, $p_{k}^{\left(0\right)}=p_{\ell }^{\left(0\right)}$. If their information sets coincide at time t, namely $I_{k}^{\left(t\right)}=I_{\ell }^{\left(t\right)}$, then* (24) *implies that their belief maps are identical.*

$$p_{k}^{\left(t\right)}=p_{\ell }^{\left(t\right)}.$$

*Thus, the belief map depends exclusively on the incorporated event set, rather than on which UAV collected the events or how they were communicated. For numerical stability, every event update is clipped to $\left[10^{-6},1-10^{-6}\right]$. Intermediate clipping can introduce order effects after a score reaches a clipping boundary, so the exact invariance statement does not cover that regime.*

Theorem 1 shows that posterior beliefs depend on the initial probability map and the accumulated information set, but not on the event-processing order. It therefore provides a theoretical foundation for distributed belief fusion. If all UAVs eventually acquire the same information set, their local belief maps converge to a common posterior without centralized coordination. This event-set viewpoint is related to communication-efficient probabilistic fusion in multisensor systems [47], while the present work specializes the exchanged information to positive detection events for distributed target search.

## 4. Distributed Selective Flooding (DSF) Consensus Algorithm

Section 3 showed that UAVs with the same prior and the same accumulated event set obtain identical belief maps. Achieving this consistency in a distributed network, however, cannot rely on exchanging full belief maps or complete detection histories. This section presents a distributed selective flooding (DSF) algorithm that propagates only positive detection events, balancing belief consistency with communication efficiency.

The algorithm operates in discrete communication rounds. Within each round, every UAV transmits its unsent positive events to each neighbor and merges received events into its local set.

Let $E_{k}^{\left(t\right)}$ denote the set of informative positive detection events held by UAV *k* up to time *t*, including events generated locally and events received from neighboring UAVs.

$$E_{k}^{\left(t\right)}=\left\{\left(n,m,\tau ,P_{D}^{\ell ,n,m}\left(\tau \right),P_{F}^{\ell ,n,m}\left(\tau \right)\right):\ell \in N_{k}\left(\tau \right),Z_{\ell ,n,m}^{\left(\tau \right)}=1,\tau \leq t\right\},$$

where $N_{k}\left(\tau \right)$ denotes the neighbor set of UAV *k* at time τ. In many search scenarios, targets are sparse and only a small fraction of grid cells are occupied. Negative detection events are therefore frequent and usually contribute limited incremental information. Transmitting positive-event innovations preserves the most informative evidence while substantially reducing communication load, which motivates a strategy in which communication is activated by new positive detections.

To support efficient information sharing, DSF implements a distributed finite-time set-consensus mechanism. Its objective is to ensure that all UAVs obtain the same set of positive detection events after finitely many neighbor-to-neighbor exchanges while avoiding full-map communication.

From the viewpoint of UAV *k*, DSF communication is needed only when UAV *k* holds positive detection events that have not yet been sent to its current neighbors. Such events may be generated by its own detector or received from other UAVs in earlier DSF exchanges. To formalize this local triggering logic, let

(25) $$\chi_{k}^{\mathrm{nbr}}\left(t\right)=\left\{\begin{array}{cc} 1, & \mathrm{if}\;N_{k}\left(t\right)\neq \emptyset ,\\ 0, & \mathrm{otherwise}, \end{array}\right.$$

denote the neighbor-based trigger, which indicates whether UAV *k* has at least one communication neighbor at time *t*. Let $t_{s-1}$ denote the most recent DSF communication instant of UAV *k*. At the next DSF opportunity $t>t_{s-1}$, define

(26) $$\Delta E_{k}^{\left(t\right)}:=E_{k}^{\left(t\right)}\setminus E_{k}^{\left(t_{s-1}\right)},$$

as the set of positive-event innovations accumulated from time $t_{s-1}$ to time *t*. This set contains new positive detections generated by UAV *k* and positive events received from other UAVs in earlier exchanges. Let

(27) $$\chi_{k}^{\det}\left(t\right)=\left\{\begin{array}{cc} 1, & \mathrm{if}\;\Delta E_{k}^{\left(t\right)}\neq \emptyset ,\\ 0, & \mathrm{otherwise}, \end{array}\right.$$

be the detection-based trigger, which indicates whether UAV *k* holds at least one unsent positive-event innovation. The DSF information-consensus trigger is then the logical conjunction of the neighbor-based and detection-based triggers.

(28) $$\chi_{k}^{\mathrm{dsf}}\left(t\right)=\chi_{k}^{\mathrm{nbr}}\left(t\right)\wedge \chi_{k}^{\det}\left(t\right).$$

After the (s−1)-th DSF communication round, let

$$t_{s}=\inf\left\{t>t_{s-1}\;|\;\chi_{k}^{\mathrm{dsf}}\left(t\right)=1\right\}$$

be the *s*-th DSF communication instant. Then, at this communication round, the local DSF update proceeds as follows.

**Remark** **2.** *Let D denote the diameter of $G_{c}$. If N≥D, then*

$$T_{k}^{\left(D\right)}=\underset{u\in U}{⋃}\Delta E_{u}^{\left(t_{s}\right)},\;\forall k\in U.$$

*Thus, after at most D rounds, every UAV obtains the union of all positive-event innovations generated within the network. However, the consensus property mentioned above requires certain conditions to be satisfied. Specifically, the undirected communication graph must remain connected throughout the N-round exchange, all nodes must participate synchronously in each round and forward newly received records even in the absence of initial innovation, every transmission must be delivered successfully without loss, and records must be compared using a unique identifier. These connectivity, synchronization, delivery, and round-budget conditions are sufficient for the guarantee, as opposed to properties that the triggering mechanism enforces by itself. Packet loss, disconnection, or an insufficient round budget invalidates the guarantee, in which case the statement describes the intended protocol behavior under ideal conditions rather than a hard execution guarantee.*

## 5. Decoupled Detection–Motion Planning Strategy

The cooperative search problem is to minimize the expected time to detect all targets, under UAV dynamics, a distance-dependent sensor (Equations (14) and (15)), and time-varying communication constraints. Because this problem is NP-hard and partially observable, computing an exact optimal policy is intractable. This section develops a structured approximation based on the time-scale separation between sensing and motion. The strategy separates search into two iterative phases. In the detection phase, a UAV remains fixed and applies a probability-priority ordering within its FoV. In the motion phase, it selects a new configuration by maximizing the Bayesian detection potential balanced against travel cost. The ordering is computationally light, while its optimality is restricted to the assumptions of Theorem 2.

The motivation is that target sensing is typically much faster than UAV motion. Continuously coupling every sensing action with immediate trajectory replanning is therefore inefficient. A UAV should instead exploit its current detection coverage before planning motion toward a new search region.

### 5.1. Phase I: Greedy Detection with Optimality Guarantee

During the detection phase, each UAV remains at a fixed planar position and detects cells within the sector-shaped FoV defined in (6). For UAV *k* at time *t*, the visible cell set is

$$F_{k}\left(t\right)=S\left(q_{k}^{\left(t\right)},\psi_{k}^{\left(t\right)}\right),$$

where $q_{k}^{\left(t\right)}$ and $\psi_{k}^{\left(t\right)}$ denote its planar position and heading angle, respectively.

Let $D_{k}\left(t\right)$ be the set of cells already detected up to time *t*. The detection phase sequentially tests a subset of undetected cells in $F_{k}\left(t\right)$ with the objective of minimizing the expected time to the first target detection. Since each detection action takes the same short interval δ, this objective is equivalent to minimizing the expected number of detection actions before a positive detection occurs.

From the visible cells, UAV *k* extracts all cells that have not yet been detected in its current responsibility region.

(29) $$C_{k}\left(t\right)=\left(F_{k}\left(t\right)\cap R_{k}\left(t\right)\right)\setminus D_{k}\left(t\right),$$

where $R_{k}\left(t\right)$ is the responsibility region for UAV *k* up to time *t* defined by (4). Thus, an FoV portion that extends beyond $R_{k}\left(t\right)$ is not inspected by UAV *k*.

For notational convenience, re-index the cells in $C_{k}\left(t\right)$ by a single index i=1,…,m.

$$C_{k}\left(t\right)=\left(\left(i_{1},j_{1}\right),\left(i_{2},j_{2}\right),\ldots \left(i_{m},j_{m}\right)\right),$$

where $m=|C_{k}\left(t\right)|$. Let $\pi =(\pi_{1},\ldots ,\pi_{m})$ be a permutation of the indices specifying the local detection sequence

$$\Pi_{k}^{\det}\left(t\right)=\left(\left(i_{\pi_{1}},j_{\pi_{1}}\right),\left(i_{\pi_{2}},j_{\pi_{2}}\right),\ldots \left(i_{\pi_{m}},j_{\pi_{m}}\right)\right),$$

which determines the order in which UAV *k* performs detection actions within its FoV. The expected time to first true-positive detection under π is

(30) $$E\left[T_{\pi }\right]=\sum_{n=1}^{m}n\delta \;p_{k,i_{\pi_{n}},j_{\pi_{n}}}^{\left(t\right)}P_{D}^{k,i_{\pi_{n}},j_{\pi_{n}}}\left(t\right)\;\prod_{l=1}^{n-1}\left(1-p_{k,i_{\pi_{l}},j_{\pi_{l}}}^{\left(t\right)}P_{D}^{k,i_{\pi_{l}},j_{\pi_{l}}}\left(t\right)\right).$$

The following theorem establishes the optimal detection ordering.

**Theorem** **2** (Optimal Detection Ordering)**.** *Suppose the belief scores and detection probabilities are fixed during the detection phase, every sensing action takes the same duration, and the detector outcomes are conditionally independent across cells. Let*

$$p_{\pi_{n}}=p_{k,i_{\pi_{n}},j_{\pi_{n}}}^{\left(t\right)}P_{D}^{k,i_{\pi_{n}},j_{\pi_{n}}}\left(t\right),\;n=1,\ldots ,m,$$

*denote the surrogate true-positive probabilities of the candidate cells in $C_{k}\left(t\right)$. The detection sequence that minimizes the expected detection time in* (30) *is obtained by ordering the cells in nonincreasing $p_{\pi_{n}}$.*

$$p_{\pi_{1}}\geq p_{\pi_{2}}\geq \ldots \geq p_{\pi_{m}}.$$

*Equivalently, the greedy rule that always detects the highest-belief candidate first is globally optimal for the stationary detection phase.*

**Proof.**  The proof uses a pairwise interchange argument. Consider an arbitrary detection sequence π and suppose that positions *r* and r+1 contain an inversion.

$$p_{\pi_{r}}<p_{\pi_{r+1}},$$

where cell $\left(i_{\pi_{r}},j_{\pi_{r}}\right)$ is detected before cell $\left(i_{\pi_{r+1}},j_{\pi_{r+1}}\right)$.

Let

$$Q=\prod_{l=1}^{r-1}\left(1-p_{\pi_{l}}\right)$$

denote the probability that no target has been detected before position *r*.

Only the contributions of cells $\left(i_{\pi_{r}},j_{\pi_{r}}\right)$ and $\left(i_{\pi_{r+1}},j_{\pi_{r+1}}\right)$ are affected by exchanging their order, while all other terms in (30) remain unchanged. The contribution of the original ordering to the expected detection time is

(31) $$\begin{array}{cc} J_{o} & =Q\left[r\delta \;p_{\pi_{r}}+\left(r+1\right)\delta \;\left(1-p_{\pi_{r}}\right)p_{\pi_{r+1}}\right]. \end{array}$$

If the two detection actions are exchanged, the corresponding contribution becomes

(32) $$\begin{array}{cc} J_{c} & =Q\left[r\delta \;p_{\pi_{r+1}}+\left(r+1\right)\delta \;\left(1-p_{\pi_{r+1}}\right)p_{\pi_{r}}\right]. \end{array}$$

The difference between the two contributions is

(33) $$\begin{array}{cc} J_{o}-J_{c} & =Q\delta \left[rp_{\pi_{r}}+\left(r+1\right)\left(1-p_{\pi_{r}}\right)p_{\pi_{r+1}}-rp_{\pi_{r+1}}-\left(r+1\right)\left(1-p_{\pi_{r+1}}\right)p_{\pi_{r}}\right]\\ \; & =Q\delta \left[p_{\pi_{r+1}}-p_{\pi_{r}}\right]. \end{array}$$

Since Q>0, δ>0, and $p_{\pi_{r+1}}>p_{\pi_{r}}$, it follows that

$$J_{o}-J_{c}=Q\delta \left(p_{\pi_{r+1}}-p_{\pi_{r}}\right)>0,$$

which implies that exchanging an inverted adjacent pair strictly decreases the expected detection time.

Starting from any feasible detection sequence, repeated adjacent exchanges eliminate all inversions and monotonically reduce the objective value. The resulting sequence satisfies

$$p_{\pi_{1}}\geq p_{\pi_{2}}\geq \ldots \geq p_{\pi_{m}},$$

and no further adjacent exchange can improve the objective. Hence, the descending-probability ordering is globally optimal.    ☐

**Remark** **3.** *Theorem 2 shows that, within a fixed FoV, the detection phase admits an exact optimal solution rather than a heuristic approximation. Consequently, the detection subproblem can be solved independently of the motion-planning subproblem, which provides the theoretical basis for the proposed detection–motion planning separation strategy.*

Therefore, UAV *k* sorts the cells in $C_{k}\left(t\right)$ in descending order of $p_{k,i,j}^{\left(t\right)}P_{D}^{k,i,j}\left(t\right)$ and detects them sequentially. Each detection outcome is immediately fused into the belief map using Algorithm 1, and positive detections are buffered for the next DSF round. The detection phase terminates when $C_{k}\left(t\right)=\emptyset$, meaning that no undetected cell remains in the current FoV. Theorem 2 guarantees that this greedy order is globally optimal during the stationary detection period.

### 5.2. Phase II: Information-Guided Motion Planning with Dynamic Responsibility Regions

After the local candidate set $C_{k}\left(t\right)$ becomes empty, UAV *k* selects a new waypoint to continue searching the remaining region. This subsection formulates motion planning as a distributed optimization problem over the responsibility regions introduced in Section 2.3. We first establish the structural properties of these regions, including partition invariance and conflict freedom. We then derive the waypoint objective from a detection-gain perspective and present the distributed motion-planning algorithm.

#### 5.2.1. Responsibility Regions

As defined in Section 2.3, the responsibility regions ${\left\{R_{k}\left(t\right)\right\}}_{k=1}^{K}$ form time-varying and pairwise disjoint subsets of the grid. Each region serves two purposes. First, it assigns each high-priority cell to a unique UAV, thereby suppressing duplicated detection decisions. Second, it decomposes the global motion-planning problem into local subproblems, since UAV *k* selects its next detection configuration using only the cells in $R_{k}\left(t\right)$, its local belief map, and information received from neighboring UAVs. The initial regions are generated by the Voronoi partition in (5), while the event-triggered update rule below allows the partition to adapt during the mission.

To characterize the remaining local search task, define the active cell set of UAV *k* within its current responsibility region as

(34) $$U_{k}\left(t\right)=R_{k}\left(t\right)\setminus D_{k}\left(t\right),$$

where $D_{k}\left(t\right)$ is the set of cells UAV *k* knows to have been inspected, either locally detected or through a valid received record. Thus, $U_{k}\left(t\right)$ consists of all undetected cells in the current responsibility region. The responsibility region $R_{k}\left(t\right)$ is said to be exhausted if $U_{k}\left(t\right)=\emptyset$, in which case no remaining undetected cell belongs to the current responsibility region of UAV *k*. This condition indicates that UAV *k* should enter a local stopping mode until the responsibility-region information changes.

#### 5.2.2. Event-Triggered Responsibility Region Adaptation

Responsibility regions are not updated continuously. Unlike the DSF trigger in Section 4, which disseminates positive detection events, the region-adaptation trigger decides whether UAV *k* should negotiate boundary-cell transfers with its current neighbors. From the viewpoint of UAV *k*, region adaptation is needed when local high-value cells have been exhausted, neighbor communication is available, and nearby high-belief cells can be requested from adjacent responsibility regions.

First, before initiating negotiation, UAV *k* constructs the candidate request set

(35) $$\begin{array}{cc} B_{k}^{\left(req\right)}\left(t\right)=\{\left(i,j\right)\in G\setminus D_{k}\left(t\right)\;| & p_{k,i,j}^{\left(t\right)}\geq \epsilon_{d},1\leq \mathrm{dist}\left(\left(i,j\right),R_{k}\left(t_{-}\right)\right)\leq a,{∥c_{ij}-q_{k}^{\left(t\right)}∥}_{2}\leq l_{\max}\}, \end{array}$$

where $t_{-}$ denotes the instant immediately before the current time *t*, $\epsilon_{d}$ is the boundary-request belief threshold, a∈N controls the boundary-layer thickness, and $l_{\max}>0$ preserves local reassignment. The boundary-layer constraint ensures that only high-belief cells sufficiently close to the current responsibility-region boundary are considered for transfer. The distance term is the Chebyshev distance from cell (i,j) to $R_{k}\left(t_{-}\right)$, i.e.,

$$\mathrm{dist}\left(\left(i,j\right),R_{k}\left(t_{-}\right)\right)=\min_{\left(n,m\right)\in R_{k}\left(t_{-}\right)}\max\{|i-n|,|j-m|\}.$$

The special case a=1 allows only immediately adjacent cells to be requested.

Next, to formalize the adaptation trigger, define

(36) $$H_{k}\left(t\right)=\left\{\left(i,j\right)\in U_{k}\left(t\right)|p_{k,i,j}^{\left(t\right)}\geq \epsilon_{d}\right\},$$

as the set of high-value undetected cells that still belong to the current responsibility region of UAV *k*. UAV *k* first exploits the cells in $H_{k}\left(t\right)$ and requests cells from neighboring regions only after this set becomes empty. Let

(37) $$\chi_{k}^{\mathrm{dep}}\left(t\right)=\left\{\begin{array}{cc} 1, & \mathrm{if}\;H_{k}\left(t\right)=\emptyset ,\\ 0, & \mathrm{otherwise}, \end{array}\right.$$

be the local high-value depletion trigger, and let

(38) $$\chi_{k}^{\mathrm{req}}\left(t\right)=\left\{\begin{array}{cc} 1, & \mathrm{if}\;B_{k}^{\left(req\right)}\left(t\right)\neq \emptyset ,\\ 0, & \mathrm{otherwise}. \end{array}\right.$$

be the request trigger. Moreover, to prevent premature negotiation while the UAV is still self-sufficient, define the local and requestable high-value masses and workloads as

$$\begin{array}{cccc} M_{k}^{\mathrm{loc}}\left(t\right) & =\sum_{\left(i,j\right)\in H_{k}\left(t\right)}p_{k,i,j}^{\left(t\right)},\; & W_{k}^{\mathrm{loc}}\left(t\right) & =\;|H_{k}\left(t\right)|,\\ M_{k}^{\mathrm{req}}\left(t\right) & =\sum_{\left(i,j\right)\in B_{k}^{\left(req\right)}\left(t\right)}p_{k,i,j}^{\left(t\right)},\; & W_{k}^{\mathrm{req}}\left(t\right) & =\;|B_{k}^{\left(req\right)}\left(t\right)|. \end{array}$$

Let $\alpha_{M}>0$ and $\alpha_{W}>0$ be fixed comparison ratios. The early workload-balancing trigger is

(39) $$\chi_{k}^{\mathrm{bal}}\left(t\right)=\left\{\begin{array}{cc} 1, & \mathrm{if}\;M_{k}^{\mathrm{loc}}\left(t\right)\leq \alpha_{M}M_{k}^{\mathrm{req}}\left(t\right)\;\mathrm{or}\;W_{k}^{\mathrm{loc}}\left(t\right)\leq \alpha_{W}W_{k}^{\mathrm{req}}\left(t\right),\\ 0, & \mathrm{otherwise}, \end{array}\right.$$

and the numerical evaluation fixes $\alpha_{M}=\alpha_{W}=1$. With these settings, $\chi_{k}^{\mathrm{bal}}\left(t\right)$ opens only when the local high-value set is empty or when the requestable neighboring mass or cell count is at least as large as the local remainder. Thus, the gate prevents negotiation while the UAV still has substantially more local high-value cells than what it could gain from a neighbor, thereby suppressing frequent low-benefit interaction. Using the neighbor-based trigger $\chi_{k}^{\mathrm{nbr}}\left(t\right)$ defined in (25), the local responsibility-region adaptation trigger is

(40) $$\chi_{k}^{\mathrm{adp}}\left(t\right)=\chi_{k}^{\mathrm{nbr}}\left(t\right)\wedge \chi_{k}^{\mathrm{dep}}\left(t\right)\wedge \chi_{k}^{\mathrm{req}}\left(t\right)\wedge \chi_{k}^{\mathrm{bal}}\left(t\right).$$

Thus, UAV *k* initiates responsibility-region negotiation only when it has at least one neighbor, its local high-value cells have been depleted, and nearby high-belief boundary cells are available for request. The adaptation is attempted if and only if

(41) $$\chi_{k}^{\mathrm{adp}}\left(t\right)=1.$$

Let $t_{s-1}$ denote the latest responsibility-region adaptation instant of UAV *k*, and let

$$t_{s}=\inf\left\{t>t_{s-1}\;|\;\chi_{k}^{\mathrm{adp}}\left(t\right)=1\right\}$$

be the time of *s*-th responsibility-region adaptation. Therefore, for all $t_{s}>t\geq t_{s-1}$, the responsibility region remains unchanged, i.e.,

(42) $$R_{k}\left(t\right)=R_{k}\left(t_{-}\right)=R_{k}\left(t_{s-1}\right),$$

When $t=t_{s}$, UAV *k* broadcasts the request set $B_{k}^{\left(req\right)}\left(t\right)$ and its location $q_{k}^{\left(t\right)}$ to all current neighbors $N_{k}\left(t\right)$. We now describe the conflict-resolution procedure from the perspective of a receiving UAV $\ell \in N_{k}\left(t\right)$. During a negotiation round, UAV *ℓ* may receive request sets from multiple neighbors. Let

(43) $$Q_{\ell }^{\left(rec\right)}\left(t\right)=\underset{k\in N_{\ell }\left(t\right)}{⋃}B_{k}^{\left(req\right)}\left(t\right),$$

denote the set of cells requested from UAV *ℓ* by its neighbors. For a cell $\left(i,j\right)\in Q_{\ell }^{\left(rec\right)}\left(t\right)\cap R_{\ell }\left(t\right)$, define the set of UAVs requesting this cell as

(44) $$K_{\ell ,(i,j)}\left(t\right)=\left\{k\in N_{\ell }\left(t\right)\;|\;\left(i,j\right)\in B_{k}^{\left(req\right)}\left(t\right)\right\}.$$

To assign cell (i,j) to exactly one requester in $K_{\ell ,(i,j)}\left(t\right)$, we introduce a deterministic owner-side tie-breaking rule. Define the travel cost from requester *k* to cell (i,j) as

(45) $$c^{\left(t\right)}\left(k,\left(i,j\right)\right)={∥q_{k}^{\left(t\right)}-c_{i,j}∥}_{2}.$$

The selected recipient of cell (i,j) is

(46) $$\kappa_{\ell }^{\left(t\right)}\left(i,j\right)=\arg\min_{k\in K_{\ell ,(i,j)}\left(t\right)}c^{\left(t\right)}\left(k,\left(i,j\right)\right),$$

The cell is assigned to the requester with the smallest travel cost, and the UAV index is used as a secondary criterion when multiple requesters have identical costs. This deterministic rule yields a unique recipient for every transferable cell.

Accordingly, the set of cells yielded by UAV *ℓ* to requester *k* is

(47) $$B_{\ell \to k}^{\left(yld\right)}\left(t\right)=\left\{\left(i,j\right)\in Q_{\ell }^{\left(rec\right)}\left(t\right)\cap R_{\ell }\left(t_{-}\right)\setminus D_{\ell }\left(t\right)\;|\;\kappa_{\ell }^{\left(t\right)}\left(i,j\right)=k\right\}.$$

Thus, UAV *ℓ* yields only requested cells that currently belong to its own responsibility region and have not yet been detected by *ℓ*. For requested cells that have already been detected by UAV *ℓ*, the corresponding historical detection records are returned to the requester.

(48) $$I_{\ell \to k}^{\left(t\right)}=\left\{\left(i,j,Z_{\ell ,i,j}^{\left(\tau \right)},P_{D}^{\ell ,i,j}\left(\tau \right),P_{F}^{\ell ,i,j}\left(\tau \right)\right)\;|\;\left(i,j\right)\in B_{k}^{\left(req\right)}\left(t\right)\cap R_{\ell }\left(t_{-}\right)\cap D_{\ell }\left(t\right),\;\kappa_{\ell }^{\left(t\right)}\left(i,j\right)=k\right\}.$$

Requester *k* then incorporates $I_{\ell \to k}^{\left(t\right)}$ into its local information set and updates its belief map using Algorithm 1, thereby avoiding redundant detection of cells whose detection histories are already available.

After all responses have been exchanged, each UAV updates its information set and responsibility region according to

(49) $$\begin{array}{cc} I_{k}^{\left(t\right)} & =I_{k}^{\left(t_{-}\right)}\cup \underset{\ell \in N_{k}\left(t\right)}{⋃}I_{\ell \to k}^{\left(t\right)}, \end{array}$$

(50) $$\begin{array}{cc} R_{k}\left(t\right) & =\left(R_{k}\left(t_{-}\right)\setminus \underset{\ell \in N_{k}\left(t\right)}{⋃}B_{k\to \ell }^{\left(yld\right)}\left(t\right)\right)\cup \underset{\ell \in N_{k}\left(t\right)}{⋃}B_{\ell \to k}^{\left(yld\right)}\left(t\right). \end{array}$$

The yielding and receiving UAVs store identical transfer records through the peer-to-peer negotiation messages. Hence, the ownership of every transferred cell is updated consistently across the communicating agents.

The responsibility-region adaptation rule has three structural properties. First, it is local because each request set in (35) is confined to a boundary layer of the current region. Second, it is event-triggered because an update occurs only when (41) is satisfied. Third, it preserves ownership because the deterministic tie-breaking rule (46) prevents simultaneous claims on the same cell. The following theorem formalizes these properties.

**Theorem** **3** (Invariance and Conflict Freedom of Responsibility Regions)**.** *Assume that the initial responsibility regions ${\left\{R_{k}\left(0\right)\right\}}_{k=1}^{K}$ are pairwise disjoint. Under the update rule* (50) *and the deterministic assignment rule* (46)*, the regions ${\left\{R_{k}\left(t\right)\right\}}_{k=1}^{K}$ remain pairwise disjoint for all t.*

(51) $$R_{k}\left(t\right)\cap R_{\ell }\left(t\right)=\emptyset ,\;\forall k\neq \ell .$$

*Consequently, no grid cell is simultaneously assigned to more than one UAV.*

**Proof.**  The proof proceeds by induction over the sequence of adaptation events. At t=0, the regions are pairwise disjoint by construction. Suppose that they are pairwise disjoint immediately before an adaptation event at time *t*. Consider an arbitrary cell g∈G. If *g* is not yielded during the event, its ownership is unchanged. If *g* is yielded, then by the induction hypothesis, it belongs to exactly one yielding region $R_{\ell }\left(t\right)$. The deterministic rule (46) assigns *g* to at most one requester, and the update (50) removes *g* from $R_{\ell }\left(t\right)$ before inserting it into the receiving region. Hence, *g* cannot belong to two updated regions simultaneously. Pairwise disjointness is preserved after the event, and the claim follows by induction.    ☐

Theorem 3 formalizes the conflict-free cell assignment achieved by the fully distributed adaptation mechanism. Although the physical fields of view of different UAVs may overlap, duplicated detection decisions are avoided because each UAV evaluates detection gain only over cells belonging to its own responsibility region.

#### 5.2.3. Waypoint Selection as Detection Gain Maximization

After heading selection, the waypoint-level gain is evaluated from the optimized visible set. At each motion-planning instant, UAV *k* selects a new waypoint from which the next stationary detection phase will be executed. Owing to the partition invariance established in Theorem 3, waypoint selection can be solved locally over the current responsibility region $R_{k}\left(t\right)$. In particular, UAV *k* evaluates only cells assigned to $R_{k}\left(t\right)$ and excludes cells that have already been detected.

Let

(52) $${\tilde{U}}_{k}\left(t\right)=\left\{\begin{array}{cc} H_{k}\left(t\right), & \mathrm{if}\;H_{k}\left(t\right)\neq \emptyset ,\\ U_{k}\left(t\right), & \mathrm{otherwise}, \end{array}\right.$$

be the waypoint-candidate base set. It consists of high-value cells in the current responsibility region when such cells exist and falls back to all remaining undetected cells only when the local high-value set is empty. Let $M_{k}\left(t\right)=\left|{\tilde{U}}_{k}\left(t\right)\right|$. Reindex the cells in ${\tilde{U}}_{k}\left(t\right)$ as

$${\tilde{U}}_{k}\left(t\right)={\left\{\left(i_{r},j_{r}\right)\right\}}_{r=1}^{M_{k}\left(t\right)}$$

such that their distances from the current UAV position satisfy

$${∥c_{i_{1},j_{1}}-q_{k}^{\left(t\right)}∥}_{2}\leq \cdots \leq {∥c_{i_{M_{k}\left(t\right)},j_{M_{k}\left(t\right)}}-q_{k}^{\left(t\right)}∥}_{2}.$$

For a prescribed integer $d\in N_{+}$, define

(53) $$W_{k}\left(t\right)=\left\{c_{i_{r},j_{r}}\;|\;1\leq r\leq \min\left\{d,M_{k}\left(t\right)\right\}\right\}.$$

Thus, $W_{k}\left(t\right)$ contains the centers of the *d* nearest high-value undetected cells whenever such cells exist in the current responsibility region, and all available candidates are retained when $M_{k}\left(t\right)<d$. If $W_{k}\left(t\right)=\emptyset$, UAV *k* has no remaining local waypoint candidate and enters the prescribed loitering mode. For a candidate waypoint $w\in W_{k}\left(t\right)$ and heading angle ψ∈[0,2π), define the set of assigned and undetected cells visible from (w,ψ) as

(54) $$A_{k}\left(w,\psi ;t\right)=S\left(w,\psi \right)\cap U_{k}\left(t\right),$$

where S(w,ψ) is the FoV cell set at position w with heading ψ as defined in (6).

For a candidate waypoint $w\in W_{k}\left(t\right)$ and heading ψ, define a surrogate probability of no true positive detection over $A_{k}\left(w,\psi ;t\right)$ for the UAV *k* as follows

(55) $${\hat{P}}_{k}^{\det}\left(w,\psi \right)=\prod_{\left(i,j\right)\in A_{k}\left(w,\psi ;t\right)}\left(1-p_{k,i,j}^{\left(t\right)}P_{D}^{k,i,j}\left(t\right)\right).$$

Thus, for any fixed waypoint $w\in W_{k}\left(t\right)$, the associated heading is selected to minimize the probability ${\hat{P}}_{k}^{\det}\left(w,\psi \right)$, i.e.,

(56) $$\psi_{k}^{⋆}\left(w\right)\in \arg\min_{\psi \in [0,2\pi )}{\hat{P}}_{k}^{\det}\left(w,\psi \right).$$

In implementation, the continuous optimization over ψ may be replaced by a finite angular grid

$$\Psi =\left\{\psi_{1},\ldots ,\psi_{N_{\psi }}\right\}\subset \left[0,2\pi \right),$$

which yields the discrete approximation

(57) $$\psi_{k}^{⋆}\left(w\right)\in \arg\min_{\psi \in \Psi }{\hat{P}}_{k}^{\det}\left(w,\psi \right).$$

The approximation error can be made arbitrarily small by refining the angular resolution, provided that the FoV boundary varies continuously with the heading except at finitely many cell-boundary events.

Given $\psi_{k}^{⋆}\left(w\right)$, define the optimized visible set

(58) $$A_{k}\left(w;t\right)=A_{k}\left(w,\psi_{k}^{⋆}\left(w\right);t\right),$$

and the corresponding detection-gain function

(59) $${\hat{P}}_{k}^{\det}\left(w\right)={\hat{P}}_{k}^{\det}\left(w,\psi_{k}^{⋆}\left(w\right)\right).$$

The value ${\hat{P}}_{k}^{\det}\left(w\right)$ measures the probability of no true positive detection during the next stationary detection phase if UAV *k* moved to w and adopted heading $\psi_{k}^{⋆}\left(w\right)$.

Although ${\hat{P}}_{k}^{\det}\left(w\right)$ favors waypoints with high immediate detection potential, it does not account for the distance required to reach them. The motion objective is therefore regularized by the distance from the current UAV position $q_{k}\left(t\right)$ to w.

(60) $$C_{k}\left(w\right)={∥q_{k}\left(t\right)-w∥}_{2},$$

The resulting utility is

(61) $$U_{k}\left(w\right)=\lambda_{G}{\hat{P}}_{k}^{\det}\left(w\right)+\lambda_{C}C_{k}\left(w\right),\;\lambda_{G},\lambda_{C}>0.$$

The coefficient $\lambda_{G}$ weights the surrogate no-detection probability, while $\lambda_{C}$ penalizes travel distance. Since only cells in $R_{k}\left(t\right)\setminus D_{k}\left(t\right)$ contribute to ${\hat{P}}_{k}^{\det}\left(w\right)$, cells already inspected by UAV *k* and cells assigned to other UAVs do not increase the utility. A cell inspected by another UAV is excluded only after an allowed DSF or region-response record has been received. The teamwide inspection union is used only for post-trial evaluation and is unavailable to the planner.

The selected waypoint and corresponding heading are

(62) $$\begin{array}{cc} w_{k}^{⋆}\left(t\right) & \in \arg\min_{w\in W_{k}\left(t\right)}U_{k}\left(w\right), \end{array}$$

(63) $$\begin{array}{cc} \psi_{k}^{⋆}\left(t\right) & =\psi_{k}^{⋆}\left(w_{k}^{⋆}\left(t\right)\right). \end{array}$$

UAV *k* then follows a feasible trajectory toward $w_{k}^{⋆}\left(t\right)$, using a straight-line path when admissible and otherwise respecting platform constraints such as acceleration limits and collision avoidance [19]. Upon arrival, UAV *k* re-enters the detection phase and sequentially detects the cells in its new FoV.

## 6. Overall Cooperative Search Algorithm

The preceding sections have developed three functional components, namely the Bayesian local belief update in Algorithm 1, the DSF-based positive-event consensus algorithm in Algorithm 2, and the responsibility-region-based motion-planning rule. We now summarize their interaction from the local viewpoint of UAV *k* in a single overall algorithm. At each iteration, UAV *k* first exploits its current FoV for local detection, invokes DSF only when positive-event innovations must be sent to current neighbors, and then executes the integrated motion-planning stage when a new detection configuration is required.
**Algorithm 2** DSF Algorithm for Event-Set Consensus from the Viewpoint of UAV *k***Require:** Local event set $E_{k}^{\left(t_{s-1}\right)}$, information set $I_{k}^{\left(t_{s-1}\right)}$, innovation set $\Delta E_{k}^{\left(t_{s}\right)}$, neighbor set $N_{k}\left(t_{s}\right)$, and maximum number of DSF exchange rounds *N*.**Ensure:** Positive detection event set $E_{k}^{\left(t_{s}\right)}$ and information set $I_{k}^{\left(t_{s}\right)}$.**Initialize:** $T_{k}^{\left(0\right)}=\Delta E_{k}^{\left(t_{s}\right)}$.**for** r=1,2,…,N **do**    **for** each neighbor $\ell \in N_{k}$ **do**        Select subset $S_{k\to \ell }^{\left(r\right)}\subseteq T_{k}^{\left(r-1\right)}$ whose elements have neither been sent to *ℓ* nor received from *ℓ*.        Transmit $S_{k\to \ell }^{\left(r\right)}$ to neighbor *ℓ*.        Receive $S_{\ell \to k}^{\left(r\right)}$ from neighbor *ℓ*.    **end for**    Update the local set according to$$T_{k}^{\left(r\right)}=T_{k}^{\left(r-1\right)}\cup \underset{\ell \in N_{k}}{⋃}S_{\ell \to k}^{\left(r\right)}$$**end for**Update $E_{k}^{\left(t_{s}\right)}\leftarrow E_{k}^{\left(t_{s-1}\right)}\cup T_{k}^{\left(N\right)}$.Update $I_{k}^{\left(t_{s}\right)}\leftarrow I_{k}^{\left(t_{s-1}\right)}\cup T_{k}^{\left(N\right)}$.

Algorithm 3 summarizes the closed-loop implementation of the proposed framework from the local viewpoint of UAV *k*. Each UAV updates its belief map after local detections or newly received events, invokes the DSF algorithm only when positive-event innovations need to be disseminated, and then performs the integrated motion-planning stage. This stage gives priority to high-value cells in the current responsibility region and activates responsibility-region adaptation only after the local high-value set has been depleted and neighboring high-belief boundary cells are available for request. If ${\tilde{U}}_{k}\left(t\right)=\emptyset$ holds after the adaptation step, UAV *k* maintains its current position until the responsibility-region information changes. Hence, the algorithm links the three designed components through local event conditions rather than centralized scheduling. The following theorem provides some theoretical properties of Algorithm 3.

**Theorem** **4** (Theoretical Guarantees of the Overall Framework)**.** *Consider Algorithm 3 under the Bayesian update rule in Algorithm 1, the DSF event-consensus algorithm in Algorithm 2, and the integrated responsibility-region-based motion-planning rule. Suppose that all UAVs are initialized with the same prior belief map, that each active DSF communication graph is connected with diameter no larger than D, and that DSF runs for at least D rounds at each activation instant. Then, the proposed framework satisfies the following properties.*

*1.*  ***Belief consistency.*** *The posterior belief is invariant to the order of locally detected and received events. Moreover, after each DSF communication, all UAVs acquire the same positive-event set in finitely many communication rounds and achieve a common posterior belief map for the disseminated information.*
*2.*  ***Optimal local detection.*** *Within each detection phase, the greedy descending-probability ordering minimizes the expected time to first target detection over the current FoV.*
*3.*  ***Conflict-free coordination.*** *If the initial responsibility regions are pairwise disjoint, then the event-triggered responsibility-region update preserves pairwise disjointness for all subsequent adaptation instants. Hence, no grid cell is simultaneously assigned to more than one UAV.*
*4.*  ***Finite-time high-belief coverage.*** *If the mission horizon $T_{\max}$ is sufficiently large and the communication graph permits information propagation, every cell whose posterior belief remains above the boundary-request threshold $\epsilon_{d}$ is either detected by its current owner or transferred through boundary-cell negotiation to a neighboring UAV in finite time.*

*Consequently, the complete framework integrates order-invariant Bayesian fusion, finite-time event-set consensus, locally optimal stationary detection, and conflict-free responsibility-region adaptation within a distributed cooperative search loop.*

**Proof.**  The belief-consistency statement follows from Theorem 1 and the finite-time event-set consensus property of DSF when the number of exchange rounds is no smaller than the graph diameter. The optimal local-detection statement follows from Theorem 2. The conflict-free coordination statement follows from Theorem 3. Finally, under a sufficiently long mission horizon, the waypoint rule in (62) repeatedly directs UAV *k* toward high-value undetected cells in its current responsibility region when such cells exist, while the adaptation trigger in (40) requests nearby high-belief boundary cells only after the local high-value set has been depleted. Hence, high-belief cells are either detected locally, transferred to a neighboring UAV through responsibility-region negotiation, or deferred until the responsibility-region information changes.    ☐

In what follows, we analyze the per-activation computational and communication costs. Let G=|G|=MN, $m_{k}\left(t\right)=\left|\Delta I_{k}^{\left(t\right)}\right|$, $n_{+}\left(t\right)$ be the number of unique positive events stored locally, $E_{c}\left(t\right)=\left|E\left(t\right)\right|$, and $N_{r}$ be the number of DSF rounds. Let $u_{k\ell }^{\left(r\right)}$ be the records sent from UAV *k* to neighbor *ℓ* in round *r*. Further, let $c_{k}\left(t\right)=\left|C_{k}\left(t\right)\right|$, $w_{k}\left(t\right)=\left|W_{k}\left(t\right)\right|$, $M_{k}\left(t\right)=\left|{\tilde{U}}_{k}\left(t\right)\right|$, $N_{\psi }=\left|\Psi \right|$, $s_{\mathrm{FoV}}=\max_{q,\psi }\left|S\left(q,\psi \right)\right|$, and let $B_{k}\left(t\right)$ denote the set of cells within Chebyshev distance [1,a] of $R_{k}\left(t\right)$. Table 2 summarizes the per-activation costs when unique-event identifiers and spatial masks are available.

Algorithm 1 evaluates each incremental event over the full grid. DSF transmits at most two directed messages per undirected link and round. Per-neighbor histories suppress echoes, so the total transmitted record volume $\sum u_{k\ell }^{\left(r\right)}$ equals initial innovations plus received records forwarded exactly once per edge. Candidate sensing cells are sorted once per stationary phase, and the outcomes per candidate contribute the $O(c_{k}\left(t\right)G)$ term. With precomputed boundary-distance and owner maps, the request set is built by traversing the cells within Chebyshev distance [1,a] of $R_{k}\left(t\right)$ and intersecting with neighbor regions, whose size is $|B_{k}\left(t\right)|=O\left(a\min\left\{M,N\right\}\right)$ for a contiguous region. Candidate waypoints are formed from ${\tilde{U}}_{k}\left(t\right)$, a subset of $R_{k}\left(t\right)$, by sorting its $M_{k}\left(t\right)$ elements rather than the full grid. Each waypoint is then evaluated over $N_{\psi }$ headings, and computing ${\hat{P}}_{k}^{\det}\left(w\right)$ from (59) multiplies at most $s_{\mathrm{FoV}}$ cell-level terms.

Without cached spatial information, each FoV evaluation requires a full-grid scan at $O(w_{k}\left(t\right)N_{\psi }G)$, direct distance-to-region evaluation costs $O(G^{2})$ per requester, and waypoint sorting degrades to O(GlogG). Precomputed FoV masks, boundary-distance transforms, and indexed region-member sets recover the lower costs in Table 2. All modules are polynomial in the grid dimensions and are performed locally per UAV under the policy specification.
**Algorithm 3** Overall Distributed Cooperative Search for UAV *k***Require:** Initial state $\left(q_{k}^{\left(0\right)},\psi_{k}^{\left(0\right)}\right)$, initial prior $p_{k}^{\left(0\right)}$, initial responsibility region $R_{k}\left(0\right)$, mission horizon $T_{\max}$.**Ensure:** Local positive-event set $E_{k}^{\left(t\right)}$, local information set $I_{k}^{\left(t\right)}$, local belief map $p_{k}^{\left(t\right)}$, and trajectory of UAV *k*.Initialize $I_{k}^{\left(0\right)}=\emptyset$, $E_{k}^{\left(0\right)}=\emptyset$, $D_{k}\left(0\right)=\emptyset$, and $p_{k}^{\left(0\right)}=p_{k}^{\left(0\right)}$.**for** $t=0,1,\ldots ,T_{\max}$ **do****Detection Stage**    Update the local neighbor set $N_{k}\left(t\right)$ by (10).    Compute $F_{k}\left(t\right)=S\left(q_{k}\left(t\right),\psi_{k}\left(t\right)\right)$ by (6) and construct $C_{k}\left(t\right)$ by (29).    **while** $C_{k}\left(t\right)\neq \emptyset$ **do**        Select the next cell in $C_{k}\left(t\right)$ according to Theorem 2.        Acquire the binary outcome *z* and form the event $e=(i,j,z,P_{D}^{k,i,j}\left(t\right),P_{F}^{k,i,j}\left(t\right))$.        Update $I_{k}^{\left(t\right)}\leftarrow I_{k}^{\left(t\right)}\cup \left\{e\right\}$ and $D_{k}\left(t\right)\leftarrow D_{k}\left(t\right)\cup \left\{\left(i,j\right)\right\}$.        **if** z=1 **then**           Update $E_{k}^{\left(t\right)}\leftarrow E_{k}^{\left(t\right)}\cup \left\{e\right\}$.        **end if**        Update $p_{k}^{\left(t\right)}$ using Algorithm 1 with the incremental event {e}.        Remove the processed (i,j) cell from $C_{k}\left(t\right)$.    **end while****Communication Stage**    Construct $\Delta E_{k}^{\left(t\right)}$ by (26) and compute $\chi_{k}^{\mathrm{dsf}}\left(t\right)$ by (28).    **if** $\chi_{k}^{\mathrm{dsf}}\left(t\right)=1$ **then**        Run Algorithm 2 with the current neighbors in $N_{k}\left(t\right)$.        Fuse newly received positive events into $I_{k}^{\left(t\right)}$ and $E_{k}^{\left(t\right)}$.        Update $p_{k}^{\left(t\right)}$ using Algorithm 1 if new events have been received.    **end if**    Construct $B_{k}^{\left(req\right)}\left(t\right)$ by (35), and compute $\chi_{k}^{\mathrm{adp}}\left(t\right)$ by (40).    **if** $\chi_{k}^{\mathrm{adp}}\left(t\right)=1$ **then**        Broadcast $\left\{B_{k}^{\left(req\right)}\left(t\right),q_{k}^{\left(t\right)}\right\}$ to all neighbors in $N_{k}\left(t\right)$.        Receive ${\left\{B_{\ell }^{\left(req\right)}\left(t\right),q_{\ell }^{\left(t\right)}\right\}}_{\ell \in N_{k}\left(t\right)}$ from all neighbors in $N_{k}\left(t\right)$.        **for** each $\ell \in N_{k}\left(t\right)$ **do**           Construct sets $B_{k\to \ell }^{\left(yld\right)}\left(t\right)$ and $I_{k\to \ell }^{\left(t\right)}$ by (47) and (48), respectively.           Broadcast $B_{k\to \ell }^{\left(yld\right)}\left(t\right)$ and $I_{k\to \ell }^{\left(t\right)}$ to its neighbor *ℓ*.           Receive $B_{\ell \to k}^{\left(yld\right)}\left(t\right)$ and $I_{\ell \to k}^{\left(t\right)}$ from its neighbor *ℓ*.        **end for**        Update $I_{k}^{\left(t\right)}$ and $R_{k}\left(t\right)$ by (49) and (50), respectively.        Update $p_{k}^{\left(t\right)}$ by Algorithm 1 if new events have been received.    **end if****Motion Planning Stage**    Construct set ${\tilde{U}}_{k}\left(t\right)$ by (52).    **if** ${\tilde{U}}_{k}\left(t\right)\neq \emptyset$ **then**        Construct set $W_{k}\left(t\right)$ by (53).        **for** each $w\in W_{k}\left(t\right)$ **do**           Compute $\psi_{k}^{⋆}\left(w\right)$ by (56) or (57).           Compute $A_{k}^{⋆}\left(w;t\right)$, ${\hat{P}}_{k}^{\det}\left(w\right)$, and $U_{k}\left(w\right)$ by (58), (60) and (61), respectively.        **end for**        Select $w_{k}^{⋆}\left(t\right)$ and $\psi_{k}^{⋆}\left(t\right)$ by (62) and (63), respectively.        Move toward $w_{k}^{⋆}\left(t\right)$ subject to platform constraints.    **else**        Maintain the current position until the responsibility-region information changes.        **break**    **end if****end for**

## 7. Numerical Experiments

This section evaluates the proposed distributed event-driven Bayesian search framework through Monte Carlo simulations. The experiments examine four aspects of the proposed design. First, spatially correlated Bayesian prediction should accelerate target detection in clustered scenes. Second, distributed selective flooding should improve team-level information consistency without full belief-map exchange. Third, dynamic responsibility-region adaptation should reduce redundant detection relative to unpartitioned greedy search. Fourth, the travel-cost weight in the waypoint utility should regulate the tradeoff between rapid detection and motion effort. Applying all comparison methods to the 100 matched scenes produces 800 trials. The parameter study evaluates 16 settings with 20 repetitions per setting, while the robustness study evaluates 11 team-size, target-number, target-layout, and larger-map cases with 20 repetitions per case. These two secondary studies contain 540 trials. In total, the evaluation comprises 1740 trials.

### 7.1. Simulation Setup

First, we introduce the scenario and UAV initialization. All principal comparisons use a 40×40 unit-cell grid with four UAVs and three stationary targets. Each case has different initial UAV states, prior hotspots, and target locations but shares one initial responsibility region. The initial communication graph is connected but not complete. The broader randomized comparison independently generates 50 additional scenes. In those scenes, one integer initial position is sampled from each of four corner-near boxes, and the four positions are assigned randomly to the UAV labels. Each initial heading points approximately toward the map center. Initial responsibility regions follow (5). A target lying in an initial FoV does not cause a scene to be discarded.

**Figure 6:**
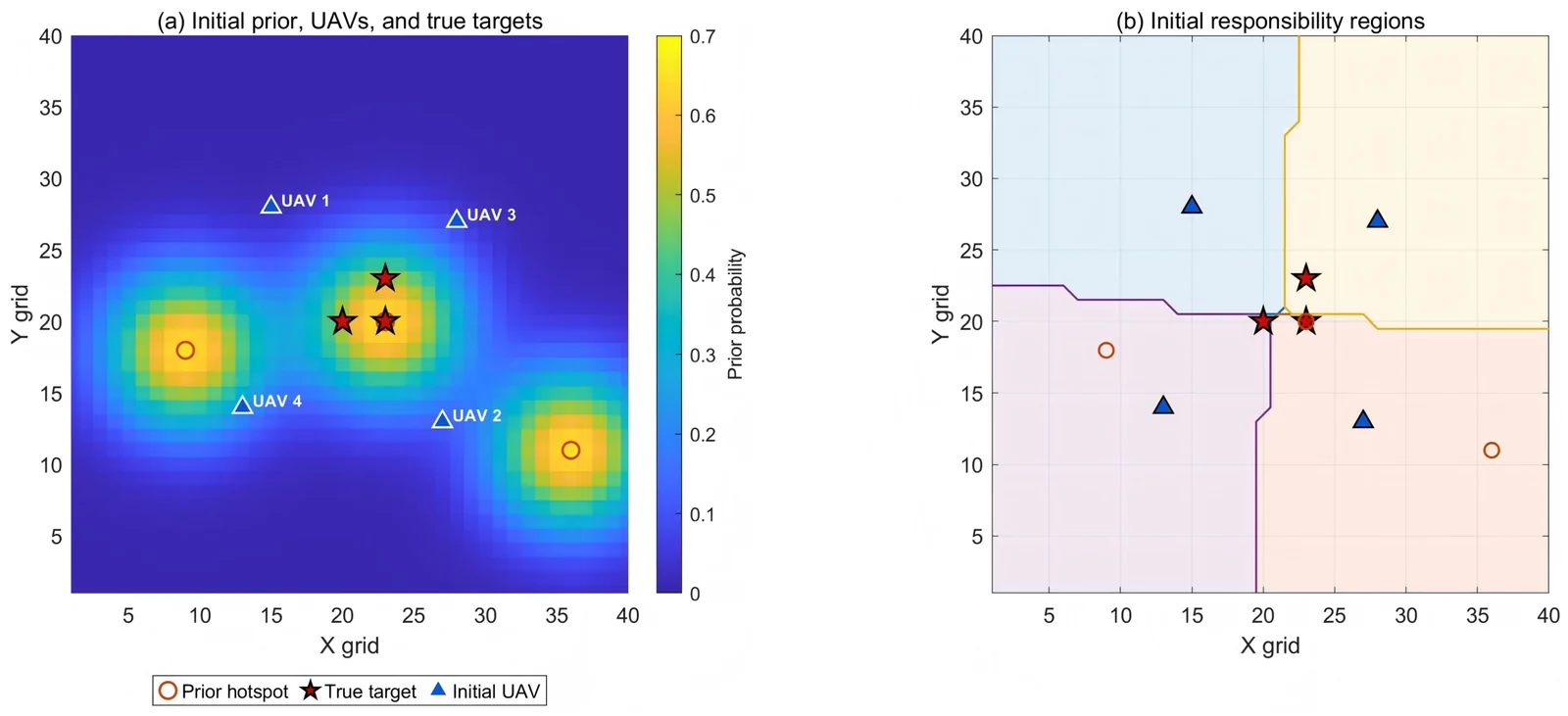
Illustration of one scenario used in the Monte Carlo simulations. In (**a**), it shows the initial prior, prior-hotspot centers, true targets, and initial UAV positions. In (**b**), the four pastel background colors identify the initial Voronoi responsibility regions owned by four different UAVs, and the colored lines mark their boundaries. The three targets occupy three initial Voronoi regions, while the three visible prior modes have equal weight and the target-generating mode is hidden from every method.

Each scene contains three equal-weight prior hotspots. In the broader randomized comparison, both integer coordinates of a hotspot center lie between 7 and 34, and the three centers have a minimum pairwise separation of 12 cells. One center generates three distinct target cells within radius 3 using weights $\exp[-d^{2}/\left(2\cdot 1.5^{2}\right)]$. The target-generating center is hidden from all methods, while the other two visible modes act as decoys. Figure 6 illustrates one scene and its initial responsibility partition. The initial prior used in the experiments is

$$p_{ij}^{\left(0\right)}=p_{\min}+\left(p_{\mathrm{peak}}-p_{\min}\right)g_{ij},$$

where $g_{ij}$ is the unit-maximum sum of the three Gaussian density functions, and $p_{\min}=0.005$ and $p_{\mathrm{peak}}=0.65$ are the minimum and maximum initial scores, respectively. No subsequent normalization is applied.

Next, we set the UAV sensing capability as follows. Every method uses the same sector sensor with detection radius $R_{\max}=6$ cells, aperture θ=π/3, and sensing duration $\tau_{s}=0.08$ s per cell. Sensing and straight-line motion durations are accumulated serially across UAVs. The detector is calibrated to achieve $P_{D}\left(R_{\max}\right)=0.95$ at the field-of-view boundary with nominal false-alarm probability $P_{F}^{⋆}=0.03$. Its detection probability varies with sensing distance according to Section 3.1. Each sensing action interrogates one visible cell and produces a binary observation. Moreover, only cells in the current region–FoV intersection are interrogated.

Finally, each scenario specifies a target layout, three visible prior hotspots, and randomized initial UAV positions and headings. All compared methods use the same scene and the same potential outcome for every labeled UAV–cell–attempt combination within a matched comparison. The main parameters are listed in Table 3. Let D(t) be the teamwide union of distinct cells that have received at least one completed observation up to time *t*, i.e.,

(64) $$D\left(t\right)=\overset{K}{\underset{k=1}{⋃}}D_{k}\left(t\right)=\left\{(i,j)\in G|a\;\mathrm{UAV}\;\mathrm{has}\;\mathrm{inspected}\;(i,j)\;\mathrm{up}\;\mathrm{to}\;\mathrm{time}\;t\right\}.$$

where $D_{k}\left(t\right)$ is the set of cells UAV *k* knows to have been detected. The common hard deadline is $T_{\mathrm{end}}=\inf\{t>0\;|\;|D\left(t\right)|/\left|G|>0.8\right\}$. A sensing action is recorded only when it finishes before the deadline, and movement is truncated to the remaining mission time. Ground truth is checked after each complete sensing, communication, planning, and motion cycle.

### 7.2. Compared Methods and Metrics

The following methods are compared.

1. **Proposed:** Bayesian belief update, DSF event consensus, high-value-prioritized dynamic region adaptation, and detection-gain waypoint selection.
2. **Static Voronoi:** The same belief update and DSF algorithm as the proposed framework, but with fixed initial responsibility regions.
3. **No-DSF:** Local Bayesian search with dynamic regions, but without DSF consensus of positive events.
4. **Global Greedy:** Detection-gain waypoint selection over the full grid without responsibility regions.
5. **Lawnmower:** Deterministic sweeping over the initial responsibility regions.
6. **Random Waypoint:** Random admissible waypoint selection.
7. **Hollinger-TRO distributed data-fusion search** [24]. The adaptation retains belief prediction, pointwise-minimum fusion, complete belief-map and planned-path exchange, implicit coordination, and a four-step receding-horizon search. Identity target dynamics are used for stationary targets, and the common sector sensor replaces the source same-cell observation model. The resulting comparison is a scenario-matched adaptation, so the source single-target guarantees do not transfer directly.
8. **Hou CVT–Auction–RHPC** [25]. The adaptation retains prior-weighted centroidal Voronoi decomposition, auction-based task assignment, the published uncertainty update, a three-step planning horizon, and the $45^{\circ }$ heading-change limit. The common obstacle-free sensing, motion, and timing conditions replace the source environment. The resulting comparison is scenario-matched rather than an exact reproduction of every source component.

To provide a balanced evaluation of detection efficiency, mission completion, motion cost, redundant coverage, and communication load, we define the following team unique-cell fraction

(65) $$C\left(t\right)=\frac{\left|D(t)\right|}{\left|G\right|}.$$

where D(t) is defined by (64). Let $C_{\mathrm{end}}=C\left(T_{\mathrm{end}}\right)$ be the final cell fraction for all compared methods. The following coverage-based discovery metrics adapt in our simulations.

(66) $$\begin{array}{cc} C_{\mathrm{first}} & =\left\{\begin{array}{cc} C(T_{\mathrm{first}}), & T_{\mathrm{first}}\leq T_{\mathrm{end}},\\ C_{\mathrm{end}}, & \mathrm{otherwise}, \end{array}\right. \end{array}$$

(67) $$\begin{array}{cc} C_{\mathrm{all}} & =\left\{\begin{array}{cc} C(T_{\mathrm{all}}), & T_{\mathrm{all}}\leq T_{\mathrm{end}},\\ C_{\mathrm{end}}, & \mathrm{otherwise}, \end{array}\right. \end{array}$$

where $T_{\mathrm{first}}$ and $T_{\mathrm{all}}$ are the time of the first true-positive discovery and the first time all actual targets have been detected, respectively. Thus, $C_{\mathrm{first}}$ and $C_{\mathrm{all}}$ are defined for every trial, and smaller values indicate earlier discovery in terms of the grid fraction inspected. The stop threshold 0.8 is the same for all methods and is applied after every completed sensing action. The empirical success probability $P_{\mathrm{succ}}$ is the fraction of Monte Carlo trials in which all targets are detected before $T_{\mathrm{end}}$. The team travel distance $L_{\mathrm{team}}$ quantifies the total motion cost of all UAVs, the duplicate-detection ratio $\rho_{\mathrm{dup}}$ measures redundant visits to already detected target cells, and the average number of transmitted event messages $N_{\mathrm{comm}}$ characterizes communication overhead. Table 4 summarizes the discovery and resource metrics.

Within each matched scenario, all methods receive the same sensor, target field, visible prior, initial UAV states, potential sensor outcomes, and strict coverage cutoff. The discovery metrics depend only on unique physical inspections and therefore do not include intervening flight durations. Selected paired contrasts are accompanied by two-sided 95% confidence intervals.

### 7.3. Baseline Comparison

Table 5 and Table 6 jointly characterize the performance profile of all eight methods. Panel A of Table 5 reports all eight methods in boundary-crossing scenes in which the three-target cluster occupies at least two initial Voronoi regions. Panel B of Table 5 reports the broader randomized scenes, while Table 6 reports the resulting motion and communication costs.

In Panel A (boundary-crossing scenes), our proposed method attained $C_{\mathrm{first}}=0.093$, compared with 0.125 for Static Voronoi and 0.165 for No-DSF. The paired differences were −0.033 with a 95% confidence interval of [−0.061,−0.004] against Static Voronoi and −0.072 with [−0.118,−0.026] against No-DSF. The Proposed method also reduced $C_{\mathrm{all}}$ relative to No-DSF by 0.117 with [−0.218,−0.017] (p=0.023). Its $C_{\mathrm{all}}$ difference from Static Voronoi was 0.023 with [−0.067,0.113] (p=0.607), which is statistically comparable. All three responsibility-region methods (Proposed, Static Voronoi, No-DSF) had $\rho_{\mathrm{rev}}=0.000$ in every trial. No cell was ever repeatedly detected by different UAVs, while the other five methods without responsibility regions had mean revisit fractions from 0.202 (Hollinger-TRO) to 0.523 (Hou). Against the heuristics that allow repeated detection by different UAVs, the Proposed method’s advantages were larger. $C_{\mathrm{first}}$ was 0.264 lower than Lawnmower with [−0.322,−0.206] ($p<10^{-11}$) and 0.145 lower than Random Waypoint with [−0.213,−0.077] ($p<10^{-4}$), while $C_{\mathrm{all}}$ was 0.292 lower than Random Waypoint with [−0.381,−0.204] ($p<10^{-7}$). These results show that the pairwise Bayesian update, DSF, and dynamic region exchange each contribute a measurable gain under the intended boundary condition. Therefore, spatially correlated belief propagation gives the Proposed method a significant first-discovery advantage over both internal baselines, DSF contributes a complete-discovery benefit over No-DSF, and the heuristics that allow cross-UAV repeated detection fall substantially behind.

The two adapted external methods discovered all targets after inspecting substantially less of the grid. In Panel A, Hollinger-TRO reached $C_{\mathrm{all}}=0.203$ and Hou 0.225, compared with the Proposed method’s 0.384 (paired differences 0.181[0.121,0.272] for Hollinger-TRO and 0.159[0.085,0.233] for Hou). The Proposed method’s $C_{\mathrm{first}}=0.093$ was statistically indistinguishable from Hollinger-TRO’s 0.088 and Hou’s 0.096. Thus, the external gap is concentrated in complete-discovery rather than first-discovery coverage. In Panel B, both external methods attained $P_{\mathrm{succ}}=1.000$ with $C_{\mathrm{all}}$ values of 0.220 (Hollinger-TRO) and 0.207 (Hou), while the Proposed method reached $C_{\mathrm{all}}=0.419$. This gap arises from architectural choices favoring discovery speed over communication economy and revisits. Specifically, Hollinger-TRO performs dense rolling-horizon implicit coordination ($8^{4}=4096$ candidate paths per step, full-map and planned-path exchange every step), Hou uses global task auction over 32 CVT subtasks followed by three-step RHPC, and both execute only one short step per cycle, creating a fine-grained sense–move–replan loop that detects targets during the route. These choices carry measurable costs. As shown in Table 6, Hollinger-TRO transmitted 1,973,411 bytes (90 times the Proposed method’s 21,833 bytes) and Hou transmitted 134,082 bytes (6 times the Proposed method’s payload), while both external methods had nonzero $\rho_{\mathrm{rev}}$ (0.202 and 0.536 in Panel B, versus $\rho_{\mathrm{rev}}=0.000$ for all three responsibility-region methods).

Our proposed method decouples detection from motion. This design eliminates the dense replanning cycle that gives the external methods their discovery advantage, but it directly produces three compensating gains that are visible in the tables. First, $\rho_{\mathrm{rev}}=0.000$ in every trial, which implies that no cell is ever repeatedly detected by different UAVs. Second, communication is event-driven. Only positive detection records and compact region-request/response messages are exchanged, yielding the 90-fold and 6-fold payload reductions. Third, the responsibility-region mechanism, combined with the workload-balancing gate $\chi_{k}^{\mathrm{bal}}\left(t\right)$, suppresses frequent negotiation and prevents cross-UAV repeated detection across team-size and layout stress tests. These three results jointly establish that the Proposed method occupies a specific point in the discovery–communication–revisit tradeoff space, rather than being dominated on every dimension.

**Figure 7:**
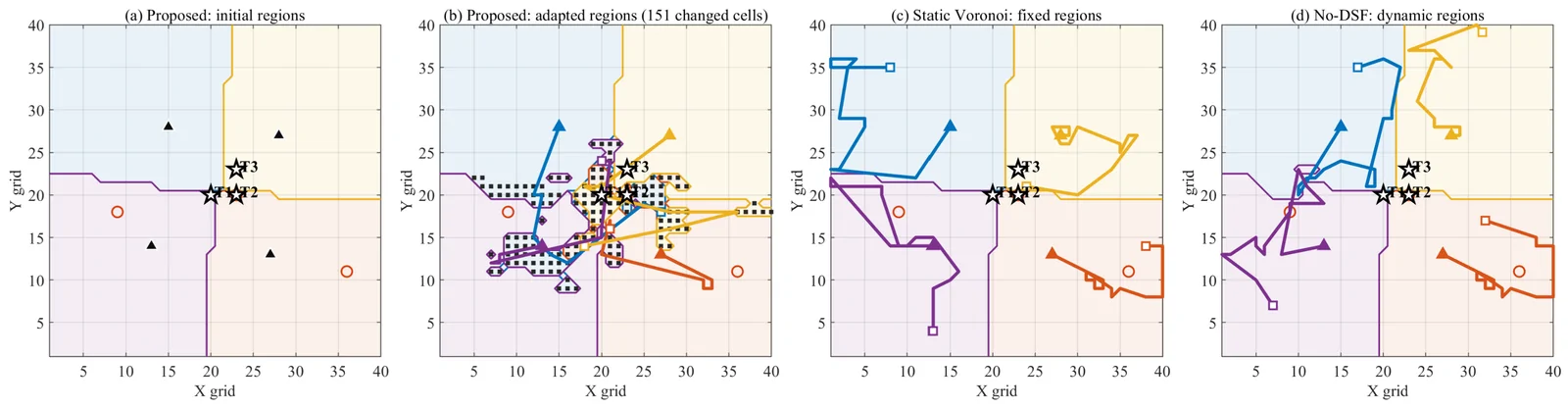
Trajectories and responsibility regions in the fixed boundary illustration. Panels (**a**–**d**) show the Proposed initial partition, Proposed adapted partition, Static Voronoi partition, and No-DSF dynamic partition, respectively. Pastel backgrounds and matching outlines denote UAV responsibility regions and their boundaries. Colored lines denote trajectories. The filled triangles and open squares mark initial and final UAV positions. Red open circles mark prior hotspots, and white stars mark targets. Black squares in panel (**b**) denote cells reassigned by adaptation. Trajectory crossings indicate route intersections, not repeated sensing or shared ownership.

**Figure 8:**
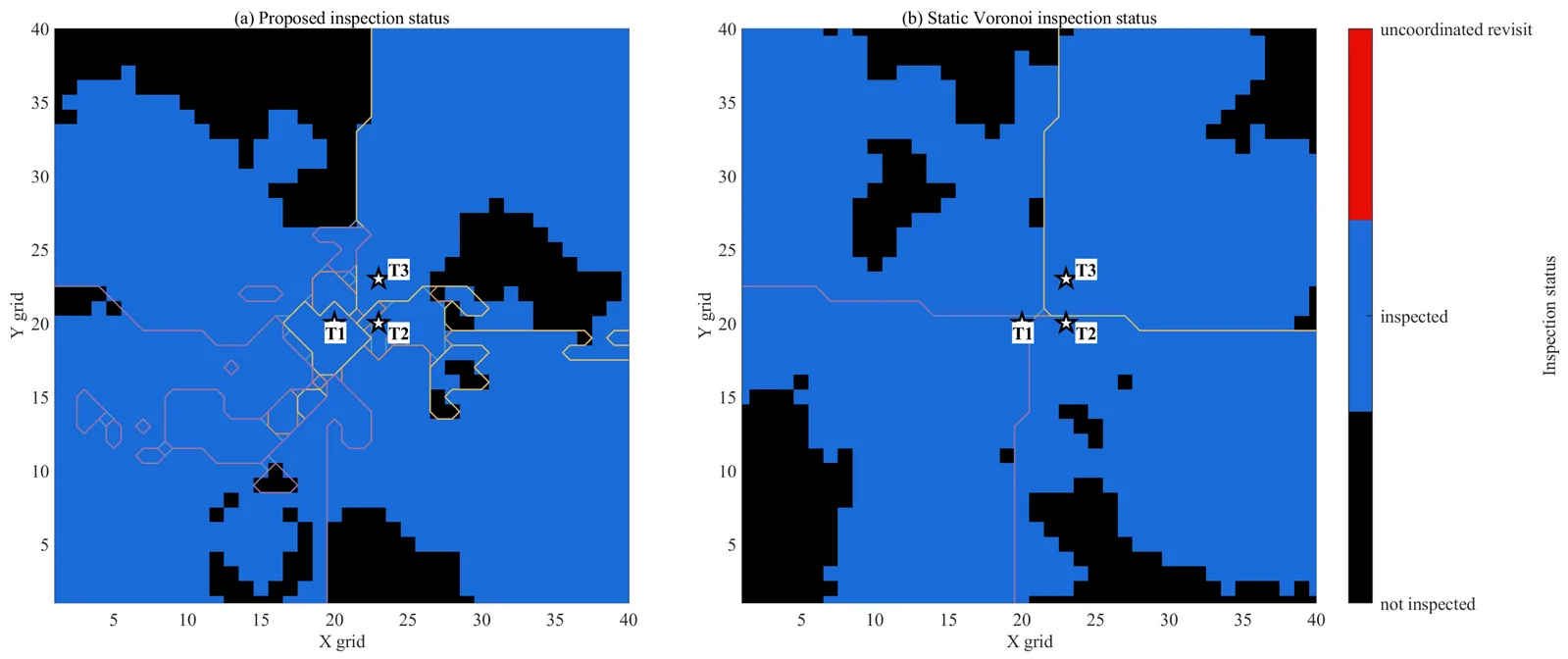
Inspection-status maps for Proposed method and Static Voronoi in the same fixed boundary illustration. Black, blue, and red denote uninspected cells, inspected cells, and uncoordinated revisits, respectively. No red cell occurs for either method. White stars labeled T1–T3 mark the true targets and are overlaid without changing the underlying cell classification. The colored lines mark the final UAV responsibility-region boundaries.

Figure 7 and Figure 8 use a single fixed boundary scene to make the mechanisms visible. Figure 7 shows that the Proposed method adapts its initial Voronoi partition through local boundary exchange, while Static Voronoi retains the fixed regions. Figure 8 confirms that the exchange produces only blue cells (each inspected once by its responsible UAV) with no red uncoordinated revisits for either the Proposed method or Static Voronoi. These qualitative observations are consistent with the $\rho_{\mathrm{rev}}=0$ result across the full 50-scene panels and are not used as quantitative evidence.

### 7.4. Positive-Event Support in the Boundary Illustration

**Figure 9:**
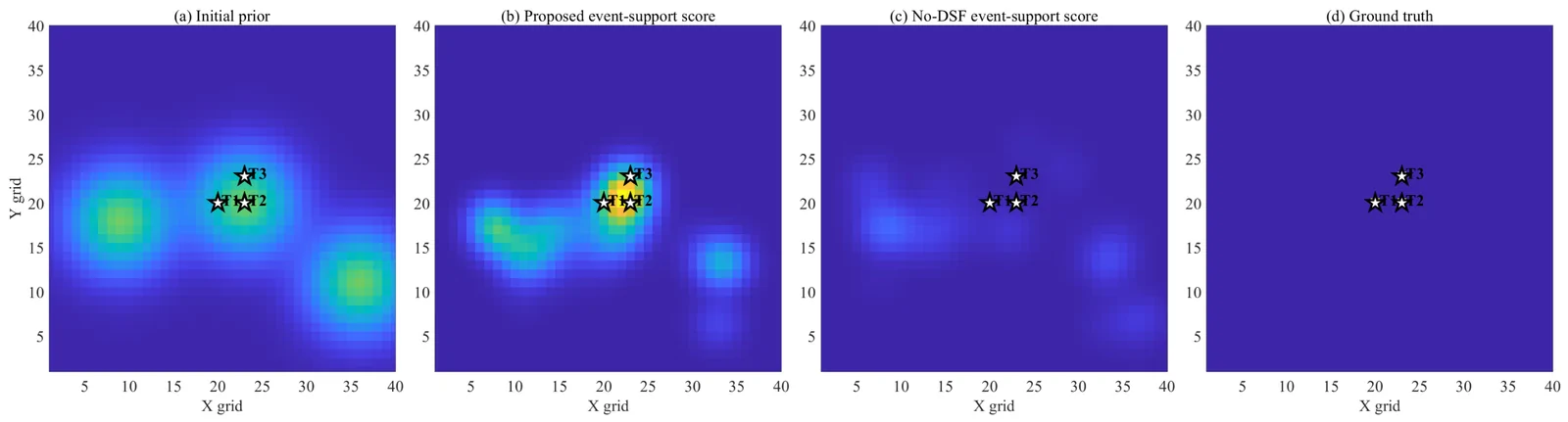
Initial prior, team-average prior-weighted positive-event support for Proposed method and No-DSF, and ground truth in the fixed boundary illustration. Under the common color scale, dark violet indicates low or zero values, blue–cyan and green indicate intermediate values, and yellow indicates high values. White stars with black outlines mark the true target locations. Their intentional overlay identifies the target coordinates and does not obscure the spatial support patterns. The common scale is determined without using the ground-truth target coordinates.

**Figure 10:**
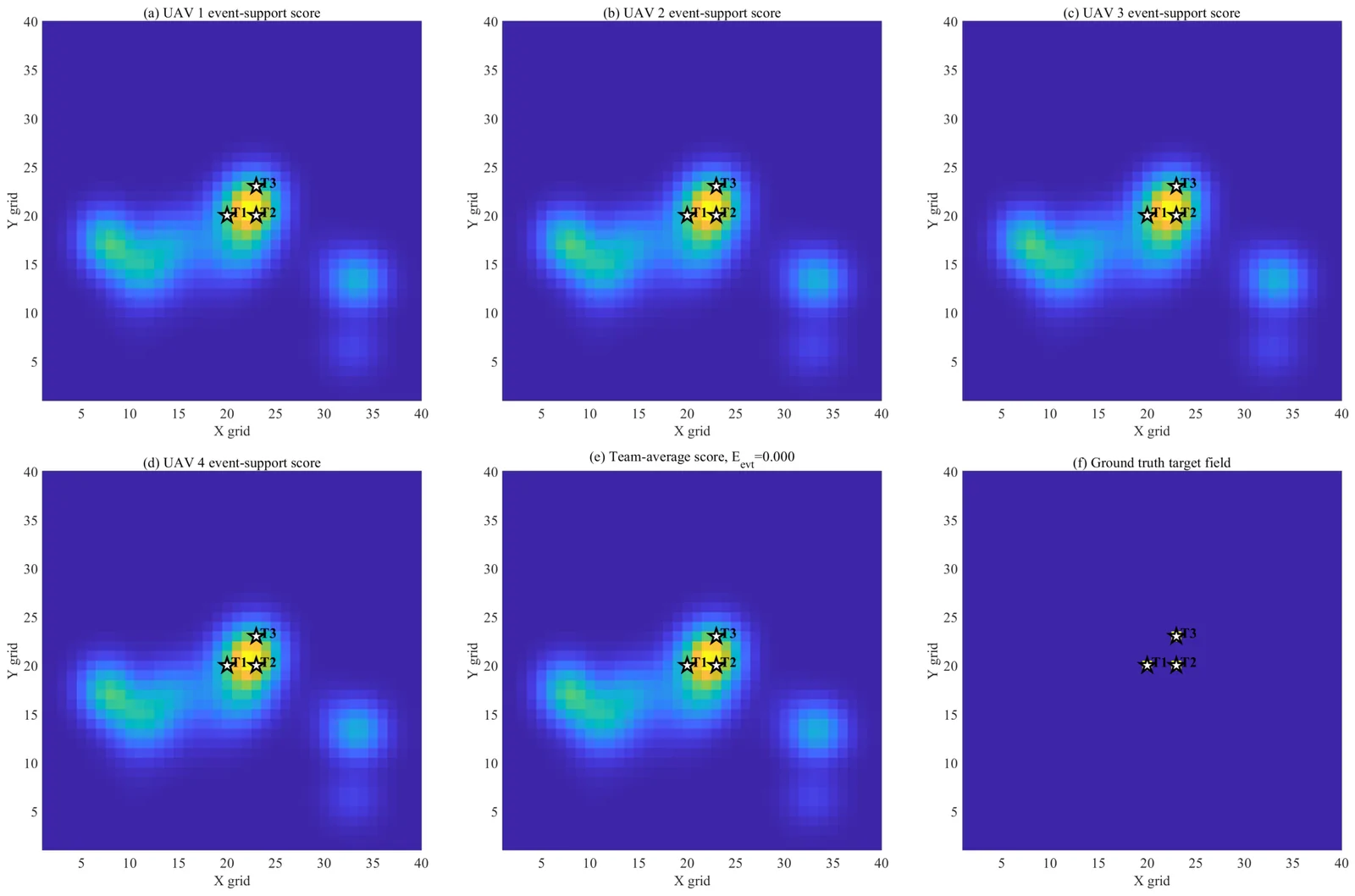
Distributed prior-weighted positive-event support in the fixed boundary illustration. The panels show the support available to the four Proposed UAVs, their team average, and ground truth. Under the common color scale, dark violet indicates low or zero support, blue–cyan and green indicate intermediate support, and yellow indicates high support. White stars with black outlines mark the true target locations.

Figure 9 and Figure 10 visualize the spatial support carried by recorded positive events. For this diagnostic only, every positive event contributes a Gaussian kernel with width $\sigma_{\mathrm{corr}}$, the summed support is weighted by the visible initial prior, and all panels use a common normalization. The maps use no ground-truth coordinate in their construction and are not substituted for the Bayesian scores used by the planner. Consequently, coherent and prior-supported positive-event clusters appear bright, most unsupported cells remain dark, and isolated false alarms remain visible rather than being removed retrospectively.

Figure 10 shows the prior-weighted positive-event support available to the four Proposed UAVs and its team average. Similar local panels indicate dissemination of the positive-event set. This does not imply equality of the complete local Bayesian maps because negative records remain local.

### 7.5. Sensitivity to Spatial-Model and Adaptation Parameters

Table 7 reports a one-factor-at-a-time sensitivity study of the correlation gain η, characteristic spacing *r*, kernel width σ, and boundary-request threshold $\epsilon_{d}$. Each setting uses 20 fixed-design repetitions; all unlisted parameters retain the default values in Table 3. The four repeated default rows (η=0.8, r=3, σ=2.2, $\epsilon_{d}=0.15$) are the same underlying trials and are not independent samples.

Increasing η from 0.5 to 3.0 changes $\left(C_{\mathrm{first}},C_{\mathrm{all}},P_{\mathrm{succ}}\right)$ from (0.202,0.296,0.95) to (0.435,0.556,0.65), identifying excessive correlation gain as the parameter with the strongest negative impact. The smallest mean $C_{\mathrm{all}}$ of 0.239 occurs at σ=1.0 with $P_{\mathrm{succ}}=0.95$. The boundary-request threshold $\epsilon_{d}$ shows nonmonotonic dependence and the highest success at 0.01 and 0.15. These are fixed-design sweeps and do not identify a generally optimal calibration.

### 7.6. Scalability and Spatial-Pattern Stress Tests

Table 8 extends the evaluation beyond the four-UAV, three-target baseline. Team-size cases place two UAVs on the horizontal midline or K≥4 UAVs uniformly on a radius-10 ring with headings toward the map center. Target-count cases distribute three, six, or nine targets among one to three local clusters. The layout cases use a compact cluster, two separated clusters, or six dispersed targets. The 60×60 farther-initialization case places four UAVs at (10,10), (50,10), (10,50), and (50,50) with a central three-target cluster and communication radius of 45. Every row uses 20 fixed-design repetitions; only the Proposed method is evaluated.

The team-size trend is nonmonotonic. Six UAVs achieve the best result in the tested ring geometry, while eight UAVs increase coordinated motion cost. The K=6 row is not covered by the finite-session consensus condition $N_{r}\geq D$ because the initial graph diameter exceeds the fixed DSF round budget. Increasing target count preserves $P_{\mathrm{succ}}=0.85$ for three, six, and nine targets, but $C_{\mathrm{all}}$ widens from 0.386 to 0.452, and $C_{\mathrm{first}}$ drops from 0.309 to near zero for nine targets because a cell very close to one initial UAV is immediately detected within the first few actions. The layout results expose the method’s dependence on clustered fields: success is 1.00 for a compact cluster, 0.55 for two clusters, and 0.45 for dispersed targets. The 60×60 case attains a success of 0.65 despite substantially farther initialization. Because every row is a fixed-design Proposed-only test, these results identify feasible regimes and failure boundaries rather than a scale-dependent advantage over a matched baseline.

### 7.7. Discussion

The coverage-based evaluation separates target discovery from motion and intentional confirmation. The Proposed method had a statistically significant first-discovery advantage over both internal baselines in boundary-crossing scenes and a complete-discovery advantage over No-DSF, while its complete-discovery coverage was comparable to Static Voronoi. In the interior controls, none of the paired internal differences was significant. In the broader randomized scenes, the Proposed method reduced complete and remaining coverage relative to Static Voronoi and was statistically comparable to No-DSF. This pattern localizes the strongest supported gain to the intended boundary condition and avoids attributing a general improvement to DSF when the broad-panel contrast does not support it.

The adapted external methods discovered all targets after inspecting less of the grid, but they did so with substantially larger application-layer payloads. Conversely, Static Voronoi and No-DSF used less travel and communication than the Proposed method by omitting region adaptation or positive-event dissemination. The results therefore show a tradeoff among discovery coverage, repeat detection by different UAVs, and communication rather than a single method dominating every metric. The sensitivity and robustness studies further show that excessive correlation gain and dispersed target layouts are the clearest tested failure regimes.

Several limitations qualify the scope of these findings.

**Communication.** The DSF finite-session consensus guarantee requires a fixed connected graph, synchronized relay participation, and successful delivery. Packet loss, delay, or disconnection invalidates it. The evaluated implementation is a synchronous reference simulator, not an asynchronous distributed deployment.

**Modeling approximations.** The detection-gain surrogate uses an independent-Bernoulli approximation. The correlated-field constant was not estimated, so the product form is a scoring surrogate rather than a calibrated probability under spatial dependence. The framework assumes stationary targets, planar motion, fixed sensor calibration, and a prescribed target count for the base prior. The one-repeat confirmation rule and workload-balancing ratios are fixed.

**Evaluation protocol.** The 0.8 stopping rule uses the teamwide inspection union, which is unavailable to individual UAVs in a deployed system. Ground truth is used only offline for discovery landmarks and $P_{\mathrm{succ}}$. The two external baselines are obstacle-free adaptations whose source guarantees do not transfer. The payload comparison excludes link-layer overhead.

Uncertain-count inference, deployable distributed termination, adaptive thresholds, and lossy-network experiments remain necessary before generalizing beyond the tested numerical study.

## 8. Conclusions

This paper developed a distributed cooperative search framework for stationary, spatially correlated targets. The framework couples three components: a pairwise Bayesian belief update that propagates detection evidence across spatially related cells, a distributed selective flooding algorithm that shares only positive detection events, and a decoupled planner that exhausts high-belief cells at a fixed position before moving, with responsibility regions dynamically renegotiated to suppress redundant coverage. The analysis establishes order-invariance of the conceptual unclipped Bayesian belief update, conditional finite-session positive-event consistency, conflict-free sensing across responsibility regions, a restricted first-pass detection-order result, and a product-form surrogate probability interpretation of the waypoint gain.

The numerical evaluation measures the unique-grid coverage required for first and complete target discovery and the coverage increment between these landmarks under a common strict 0.8 cutoff. Flight intervals are excluded from these discovery metrics, while team travel and application-layer communication are analyzed as separate resource costs. In boundary-crossing scenes, the Proposed method achieved earlier first discovery than Static Voronoi and No-DSF and lower complete-discovery coverage than No-DSF, while remaining comparable to Static Voronoi on complete discovery. The internal methods were statistically similar in the interior controls. In broader randomized scenes, the Proposed method improved complete and remaining coverage over Static Voronoi and remained comparable to No-DSF. The external adaptations achieved lower discovery coverage but required substantially larger communicated payloads. The secondary studies identified excessive correlation gain and spatially dispersed targets as the principal tested failure regimes. These results support the proposed mechanism under its intended boundary condition while making no claim of superiority on every metric or against every external method.

Future work will extend the event model to moving targets, incorporate three-dimensional terrain, obstacles, and collision constraints, replace the evaluation-layer global coverage cutoff with a deployable distributed coverage-estimation and termination rule, and evaluate the per-neighbor DSF and strictly local region trigger under packet loss, delay, asynchronous execution, and intermittent connectivity. The present two external-baseline comparisons will be broadened to additional MPC, reinforcement-learning, and swarm-intelligence methods.

## Figures and Tables

**Table 1:** Qualitative comparison with representative multi-UAV search and coordination approaches. Each column identifies a design property that, when absent, creates one of the three bottlenecks discussed in the surrounding text. **Bold** entries distinguish the proposed framework.

Approach	Online Evidence Update	Spatial Target Correlation	Communication Pattern	Detection–Motion Coupling	Responsibility-Region Coordination
MPC/DMPC search [15,23]	Grid-based recursive Bayesian update	Independent cell assumption	Full or compressed map exchange	Coupled in single receding horizon	Static Voronoi partition or none
Distributed data-fusion search [24]	Capture-state intensity Bayesian update	Independent cell assumption	Full belief-map and planned-path exchange	Single integrated planning cycle	Not incorporated
RL-based search [33,34,35]	Learned value or policy network	Independent cell assumption ^a^	Architecture-dependent	Architecture-dependent	Architecture-dependent
Swarm-intelligence search [42,43]	Heuristic-based iterative planning	Independent cell assumption	Global or regional information required	Single optimization pass	Predefined partition or none
**Proposed framework**	**Pairwise Bayesian belief update**	**Distance-dependent pairwise coupling**	**Positive-event DSF with region negotiation**	**Two-phase decoupled detection then motion**	**Event-triggered boundary-cell transfer**

^a^ Refs. [34,35] address moving or movable targets; the present work assumes stationary targets.

**Table 2:** Per-activation complexity.

Module	Computation	Storage or Communication
Bayesian belief update	$O\;\left(G\;m_{k}\left(t\right)\right)$	$O(G+n_{+}\left(t\right))$ storage
DSF activation	$O\;\left(\sum_{k,\ell ,r}u_{k\ell }^{\left(r\right)}\right)$ processing	$\leq 2N_{r}E_{c}\left(t\right)$ messages, $\sum_{k,\ell ,r}u_{k\ell }^{\left(r\right)}$ records
Greedy FoV sensing	$O\;\left(c_{k}\left(t\right)\log c_{k}\left(t\right)+c_{k}\left(t\right)G\right)$	$O(G+c_{k}\left(t\right))$ storage
Boundary request	$O(|B_{k}\left(t\right)|)$	O(G) storage
Region negotiation	O(KG)	$\leq n_{\mathrm{req}}+n_{\mathrm{grant}}+6n_{\mathrm{ret}}$ values
Waypoint selection	$O\;(|R_{k}\left(t\right)|+M_{k}\left(t\right)\log M_{k}\left(t\right)$ $+w_{k}\left(t\right)N_{\psi }s_{\mathrm{FoV}})$	$O(G+w_{k}\left(t\right))$ storage

**Table 3:** Main simulation parameters.

Parameter	Description	Value
$N_{x}\times N_{y}$	Grid size	40×40
*K*	Number of UAVs	4
$N_{T}$	Number of targets	3
$R_{\max}$	Detection radius	6 cells
$\beta_{s}$	Sensor amplitude-decay exponent	2.0
$d_{\min}$	Minimum effective sensing distance	0.5 cell
$A_{s}/\sigma_{n}$	Signal/noise calibration	$6^{2}\left[Q^{-1}\left(0.03\right)-Q^{-1}\left(0.95\right)\right]$
θ	Detection aperture	π/3
$v_{\max}$	Maximum UAV speed	1 cell/s
$\tau_{s}$	Atomic sensing duration	0.08 s
$\epsilon_{d}$	Boundary-request belief threshold	0.15
$\epsilon_{c}$	Confirmation belief threshold	0.15
η	Spatial-correlation gain	0.8
*r*	Characteristic target spacing	3 cells
σ	Spatial-kernel width	2.2 cells
$\sigma_{h}$	Prior-hotspot Gaussian width	4 cells
$p_{b}$	Pairwise base-occupancy reference	$1-{\left(1-1/1600\right)}^{3}$
$l_{\max}$	Maximum request distance	12 cells
*a*	Boundary-layer thickness	2
$\alpha_{M}$	Early mass and workload ratios	1
$\alpha_{W}$	Early mass and workload ratios	1
*d*	Number of nearest waypoint candidates	20
$\lambda_{G}$	Detection-gain weight	1
$\lambda_{C}$	Travel-cost weight	0.2
$N_{\psi }$	Candidate headings per waypoint	16
$P_{D}\left(R_{\max}\right)$	Detection probability at the FoV boundary	0.95
$P_{F}^{⋆}$	Nominal false-alarm probability	0.03
$d_{\mathrm{com}}$	Communication radius	15 cells
$N_{r}$	DSF rounds per activation	2
$N_{\mathrm{scene}}$	Pre-generated randomized scenarios	50

**Table 4:** Definitions of the comparison metrics.

Metric	Definition	Preferred
$C_{\mathrm{first}}$	Team unique-cell coverage fraction at the first true-positive target discovery, with $C_{\mathrm{end}}$ assigned when no target is discovered	Smaller
$C_{\mathrm{all}}$	Team unique-cell coverage fraction when all targets have first been detected, with $C_{\mathrm{end}}$ assigned to an incomplete trial	Smaller
$P_{\mathrm{succ}}$	Fraction of trials in which all actual targets are detected by $s_{\mathrm{end}}$	Larger
$L_{\mathrm{team}}$	Sum of the distances traveled by all UAVs up to the common coverage endpoint	Smaller
$\rho_{\mathrm{rev}}$	Fraction of cells that are repeatedly detected by different UAVs	Smaller
$N_{\mathrm{msg}}$	Number of directed application-layer search messages	Smaller
$B_{\mathrm{payload}}$	Application-layer search payload delivered to receivers in bytes	Smaller

**Table 5:** Coverage-based comparison of all eight methods over 50 boundary-crossing scenes and 50 broader randomized scenes. Coverage quantities are mean ± sample standard deviation over all trials; success probabilities include Wilson 95% confidence intervals.

Method	$C_{\mathrm{first}}$	$C_{\mathrm{all}}$	$P_{\mathrm{succ}}$ [95% CI]	$\rho_{\mathrm{rev}}$
**Panel A. Boundary-crossing scenes**				
**Proposed**	0.093±0.089	0.384±0.264	0.880[0.741,0.942]	0.000±0.000
Static Voronoi	0.125±0.122	0.391±0.249	0.840[0.715,0.917]	0.000±0.000
No-DSF	0.165±0.201	0.531±0.258	0.660[0.522,0.776]	0.000±0.000
Global Greedy	0.137±0.141	0.329±0.157	1.000[0.929,1.000]	0.477±0.087
Lawnmower	0.357±0.222	0.529±0.131	0.960[0.865,0.989]	0.411±0.013
Random Waypoint	0.238±0.232	0.707±0.168	0.420[0.294,0.558]	0.395±0.026
Hollinger-TRO	0.088±0.061	0.203±0.080	1.000[0.929,1.000]	0.226±0.094
Hou CVT–Auction–RHPC	0.096±0.084	0.225±0.138	0.980[0.895,0.996]	0.523±0.052
**Panel B. Broader randomized scenes**				
**Proposed**	0.174±0.177	0.419±0.232	0.860[0.738,0.930]	0.000±0.000
Static Voronoi	0.180±0.185	0.531±0.246	0.780[0.648,0.872]	0.000±0.000
No-DSF	0.178±0.179	0.433±0.243	0.860[0.738,0.930]	0.000±0.000
Global Greedy	0.214±0.178	0.400±0.195	0.960[0.865,0.989]	0.434±0.136
Lawnmower	0.409±0.272	0.546±0.231	0.780[0.648,0.872]	0.392±0.023
Random Waypoint	0.335±0.260	0.615±0.232	0.640[0.501,0.759]	0.393±0.021
Hollinger-TRO	0.117±0.069	0.220±0.093	1.000[0.929,1.000]	0.202±0.084
Hou CVT–Auction–RHPC	0.123±0.069	0.207±0.084	1.000[0.929,1.000]	0.536±0.045

**Table 6:** Team motion and application-layer communication over 50 randomized scenes at the common strict 0.8 unique-coverage endpoint. Entries are mean ± sample standard deviation, and communication entries exclude link-layer overhead.

Method	$L_{\mathrm{team}}$	$N_{\mathrm{msg}}$	Payload Values	$B_{\mathrm{payload}}$ (Bytes)
**Proposed**	430.6±97.8	130.8±72.2	2729.1±1594.7	21,833.0 ± 12,757.9
Static Voronoi	313.8±29.4	47.5±24.3	930.6±331.5	7444.8±2652.4
No-DSF	292.1±25.9	3.9±4.4	58.8±85.4	470.6±683.2
Global Greedy	381.1±100.4	0.0±0.0	0.0±0.0	0.0±0.0
Lawnmower	1028.5±114.3	0.0±0.0	0.0±0.0	0.0±0.0
Random Waypoint	3320.8±237.4	0.0±0.0	0.0±0.0	0.0±0.0
Hollinger-TRO	229.8±35.0	153.1±51.2	246,676.3 ± 82,557.2	1,973,410.6 ± 660,458.0
Hou CVT–Auction–RHPC	381.3±54.2	981.4±138.0	16,760.3 ± 2122.6	134,082.2 ± 16,981.0

**Table 7:** One-factor-at-a-time parameter sensitivity over 20 fixed-design trials per setting under the common 0.8 coverage cutoff. Coverage quantities are mean ± sample standard deviation; success probabilities include Wilson 95% confidence intervals.

Parameter	Value	$C_{\mathrm{first}}$	$C_{\mathrm{all}}$	$P_{\mathrm{succ}}$ [95% CI]
η	0.5	0.202±0.159	0.296±0.200	0.950[0.764,0.991]
	0.8	0.309±0.221	0.386±0.246	0.850[0.640,0.948]
	1.5	0.389±0.253	0.426±0.263	0.800[0.584,0.919]
	3.0	0.435±0.267	0.556±0.253	0.650[0.433,0.819]
*r*	2	0.203±0.133	0.404±0.261	0.800[0.584,0.919]
	3	0.309±0.221	0.386±0.246	0.850[0.640,0.948]
	5	0.260±0.234	0.358±0.276	0.800[0.584,0.919]
	8	0.100±0.020	0.285±0.290	0.800[0.584,0.919]
σ	0.5	0.173±0.102	0.312±0.288	0.900[0.699,0.972]
	1.0	0.184±0.167	0.239±0.217	0.950[0.764,0.991]
	2.2	0.309±0.221	0.386±0.246	0.850[0.640,0.948]
	3.0	0.282±0.192	0.373±0.235	0.900[0.699,0.972]
$\epsilon_{d}$	0.01	0.277±0.219	0.372±0.235	0.900[0.699,0.972]
	0.05	0.330±0.244	0.393±0.273	0.750[0.531,0.888]
	0.10	0.245±0.211	0.370±0.289	0.750[0.531,0.888]
	0.15	0.309±0.221	0.386±0.246	0.850[0.640,0.948]

**Table 8:** Scalability and spatial-pattern stress tests for the Proposed method over 20 fixed-design trials per row under the common 0.8 coverage cutoff. Coverage quantities are mean ± sample standard deviation; success probabilities include Wilson 95% confidence intervals.

Factor	Case	$C_{\mathrm{first}}$	$C_{\mathrm{all}}$	$P_{\mathrm{succ}}$ [95% CI]
Team size	K=2	0.013±0.000	0.521±0.280	0.750[0.531,0.888]
	K=4	0.128±0.034	0.260±0.199	0.900[0.699,0.972]
	K=6	0.181±0.175	0.333±0.244	0.950[0.764,0.991]
	K=8	0.227±0.106	0.410±0.240	0.800[0.584,0.919]
Target count	$N_{T}=3$	0.309±0.221	0.386±0.246	0.850[0.640,0.948]
	$N_{T}=6$	0.120±0.059	0.452±0.219	0.850[0.640,0.948]
	$N_{T}=9$	0.001±0.000	0.391±0.207	0.850[0.640,0.948]
Six-target layout	Compact cluster	0.058±0.026	0.168±0.072	1.000[0.839,1.000]
	Two clusters	0.154±0.073	0.611±0.209	0.550[0.342,0.742]
	Dispersed	0.062±0.001	0.753±0.080	0.450[0.258,0.658]
Larger/far map	60×60, $N_{T}=3$	0.021±0.000	0.317±0.365	0.650[0.433,0.819]

## Data Availability

The original contributions presented in this study are included in the article. Further inquiries can be directed to the corresponding author.
